# Novel Carbazole–Acylthiourea Derivatives: Synthesis, Antimicrobial, Antioxidant, Hemocompatibility, Cytotoxicity, and ADME‐Tox Profiling

**DOI:** 10.1002/open.70258

**Published:** 2026-10-12

**Authors:** Carmen Limban, Ioana Cristina Marinaş, Mariana Carmen Chifiriuc, Miron Teodor Căproiu, Florea Dumitraşcu, Erzsébet‐Eleonóra Kapronczai, Irina Zarafu, Diana Camelia Nuţă, Petre Ioniţă, Speranţa Avram, Cătălina Mareş, Andra Maria Păun, Bogdan Mihai Cristea, Carmellina Daniela Bădiceanu, Ilinca Margareta Vlad, Maria Coandă, Delia Laura Popescu, Coralia Bleotu

**Affiliations:** ^1^ Department of Pharmaceutical Chemistry Faculty of Pharmacy “Carol Davila” University of Medicine and Pharmacy Bucharest Romania; ^2^ Research Institute of the University of Bucharest (ICUB) University of Bucharest Bucharest Romania; ^3^ Department of Microbiology Faculty of Biology University of Bucharest Bucharest Romania; ^4^ Romanian Academy Bucharest Romania; ^5^ “C.D. Nenitzescu” Institute of Organic and Supramolecular Chemistry of the Romanian Academy Bucharest Romania; ^6^ Supramolecular Organic and Organometallic Chemistry Centre Department of Chemistry Faculty of Chemistry and Chemical Engineering Babeş‐Bolyai University Cluj‐Napoca Romania; ^7^ Department of Organic Chemistry, Biochemistry and Catalysis Faculty of Chemistry University of Bucharest Bucharest Romania; ^8^ Department of Anatomy, Animal Physiology, and Biophysics Faculty of Biology University of Bucharest Bucharest Romania; ^9^ Preclinical Department 2 Morphological Sciences Faculty of Medicine Carol Davila University of Medicine and Pharmacy Bucharest Bucharest Romania; ^10^ Faculty of Interdisciplinary Studies University of Bucharest Bucharest Romania; ^11^ Department of Cellular and Molecular Pathology Ştefan S. Nicolau Institute of Virology Bucharest Romania

**Keywords:** acylthiourea, antimicrobial resistance, carbazole, carprofen, hemocompatibility

## Abstract

A focused series of four carprofen‐derived carbazole–acylthiourea *N*‐(arylcarbamothioyl)propanamides (**1a–d**) was synthesized by in situ generation of 2‐(6‐chloro‐9*H*‐carbazol‐2‐yl)propanoyl isothiocyanate followed by reaction with aniline, *para*‐toluidine, *para‐*bromoaniline, and *para‐*iodoaniline, respectively. The structures were supported by FT‐IR, NMR, and APCI‐HRMS analyses. The compounds were evaluated against clinically relevant bacterial and fungal strains, complemented by antioxidant assays, hemocompatibility/antihemolysis models, cancer cell viability assays, and in silico drug‐likeness, pharmacokinetic, pharmacogenomic, and toxicity prediction. The biological profile was substituent‐dependent: **1b** showed the strongest activity against *Staphylococcus aureus* (MIC = 0.25 mg/mL), **1a** and **1c** were the most active against *Enterococcus faecalis* (MIC = 0.31 mg/mL), while the iodophenyl derivative **1d** was the most potent against *Candida albicans* (MIC = 0.16 mg/mL). Compounds **1b** and **1c** also inhibited microbial adherence in selected Gram‐positive and fungal models. Compound **1a** displayed the most consistent antioxidant activity across antioxidant assays and protected erythrocytes from AAPH‐induced oxidative hemolysis. The cytotoxicity screening revealed increased sensitivity of A‐431 cells, particularly to **1b** (IC50 = 13.67 µg/mL). Molecular docking revealed key binding interactions potentially underlying the observed biological activity. Overall, the integrated data identify carbazole–acylthiourea propanamides, especially **1a** and **1b**, as promising bioactive scaffolds for further optimization as multifunctional compounds.

## Graphical abstract

We synthesized four novel carprofen‐derived carbazole–acylthiourea propanamides with selective antimicrobial activity against Staphylococcus aureus, Enterococcus faecalis, and Candida albicans. Compound **1a** provides combined antioxidant and antihemolytic protection. Meanwhile, compound **1b** exhibits the highest cytotoxicity against the A‐431 epidermoid carcinoma cell line, highlighting their multifunctional therapeutic potential.

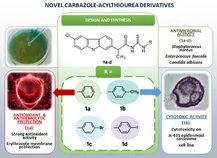

## 1 Introduction

One of the challenges of modern medicine is antimicrobial resistance (AMR) because it reduces treatment effectiveness and contributes significantly to morbidity and mortality associated with bacterial and fungal infections. The emergence of multidrug‐resistant strains requires the identification of new antimicrobial agents with innovative structures, complementary biological profiles, and potentially distinct mechanisms of action, able to address infection and infection‐associated oxidative damage [1, 2].

Carbazole and acylthiourea derivatives are attractive candidates for the development of new antibacterial agents, as their diverse mechanisms of action may help overcome bacterial drug resistance. The antimicrobial relevance of the carbazole framework has been emphasized by recent analyses of natural and semisynthetic carbazole alkaloids [3].

Carbazole‐containing compounds may increase membrane permeability by inhibiting specific enzymatic processes and therefore represent a promising strategy for persistent infections [4], while some studies have demonstrated the beneficial influence of this pharmacophore on the occurrence of an antioxidant effect [5, 7]. They may also interfere with bacterial DNA replication through interactions with DNA gyrase [8] or DNA polymerase [9].

Numerous compounds containing the propanoic acid moiety are widely recognized for potent biological activities, including antimicrobial [10], anticancer [11], and antioxidant [12] activities. Several propanoic acid derivatives are well‐known drugs with anti‐inflammatory properties, including ibuprofen, naproxen, ketoprofen, flurbiprofen, fenoprofen, pranoprofen, tiaprofenic acid, suprofen, and oxaprozin.

Carprofen, 2‐(6‐chloro‐9*H*‐carbazol‐2‐yl)propanoic acid (IUPAC), is a nonsteroidal anti‐inflammatory, analgesic, and antipyretic drug used primarily for the management of pain and inflammation in animals, particularly dogs. It belongs to the class of drugs known as cyclooxygenase (COX) inhibitors, which reduce the production of prostaglandins involved in the inflammatory and painful process. Despite its valuable pharmacological activities, it was withdrawn from the market for commercial reasons. However, the carprofen molecule continues to remain in the research focus for discovering new applications while searching for new potential biological targets or reintroducing it into human use as a modified molecule. Molecular modeling studies have indicated that carprofen derivatives have anti‐inflammatory [13, 16], antimicrobial [17, 20], antiviral [21], anticancer [22, 24], and anti‐Alzheimer [25] potential. Therefore, these scaffolds may be employed for the development of compounds with optimized pharmacological properties.

Literature data suggest that certain nonsteroidal anti‐inflammatory drugs, such as carprofen, represent promising candidates for the development of dual‐action agents for the potential treatment of coexisting inflammatory and infectious diseases, such as tuberculosis, urinary tract infections, and musculoskeletal infections [26, 27].

Acylthiourea derivatives are important in medicinal chemistry because they have been found to exhibit diverse biological activities. These derivatives are potent antimicrobial [28, 30], anticancer [31, 33], antifungal against plant pathogenic fungi [34, 35], herbicidal [36], antiangiogenic [37], and antioxidant [38, 39] agents. Also, some acylthiourea derivatives with antimicrobial activity and protective effect of host cells from oxidative stress have been synthesized [40, 41].

Acylthiourea derivatives may exert antimicrobial activity by acting at the level of the genetic material, inhibiting DNA gyrase or topoisomerase IV through metal‐ion complexes [42], at the level of cellular structures, by disrupting the cell membrane or the cell wall via interactions of the C=S, C=O and NH groups with carboxyl and phosphate groups on the bacterial membrane surface [43] and at the level of microbial energy metabolism, or by disturbing NAD+/NADH homeostasis [44]. They may also inhibit enzymes such as enoyl–acyl carrier protein reductase in *Mycobacterium tuberculosis* [45], urease [46], and SecA ATPase [47].

The thiourea fragment and the carbazole nucleus have also attracted considerable attention in medicinal chemistry due to their potential anticancer properties.

Carprofen can increase the expression of the p75 neurotrophin receptor (p75NTR) and inhibit cell survival by apoptosis through the p38 mitogen‐activated protein kinase (p38 MAPK) pathway in prostate cancer cell lines [22]. A carprofen analog, 2‐(6‐chloro‐9*H*‐carbazol‐2‐yl)propanamide, proved to be an efficient inhibitor of both SIRT1 and SIRT2, supporting the development of a new class of compounds with anticancer effects [23]. Carbazole arylsulfonamides, such as 2‐(6‐chloro‐9*H*‐carbazol‐2‐yl)‐*N*‐(4‐(*N*‐(2‐(trifluoromethyl)phenyl)sulfamoyl)phenyl)propanamide, showed good in vitro cytotoxic activity against A875, HepG2, and MARC145 cell lines [48]. Carbazole arylhydrazone derivatives also produced inhibitory effects against 7901 and A875 cancer cells, with 2‐(6‐chloro‐9*H*‐carbazol‐2‐yl)‐*N*′‐(2‐hydroxy‐3‐methoxybenzylidene)propanehydrazide showing high inhibitory activity [49].

The thiourea pharmacophore, through its C=S group acting as a hydrogen‐bond acceptor and its N—H group acting as a hydrogen‐bond donor, plays a key role in enzyme and receptor binding, thereby stabilizing ligand–receptor interactions. This feature is relevant for the design of novel anticancer agents. The antineoplastic activity of the tenovin family, including p53 activators and sirtuin (SirT) inhibitors, has further stimulated interest in thiourea derivatives as potential anticancer chemotherapeutics [50].

Therefore, we aimed to obtain novel and more effective compounds active against resistant pathogenic bacteria and fungi and through antioxidant properties, able to modulate oxidative stress occurring during infection and thus support host recovery. In addition, the strategic integration of the carbazole nucleus and the thiourea and propionic acid moieties offers great potential for the development of multifunctional compounds which can be particularly valuable in addressing complex pathological conditions where oxidative stress, infection, and tumor development are interconnected.

## 2 Results and Discussion

### 2.1 Chemistry and Structural Characterization

The novel *N*‐(arylcarbamothioyl)propanamides (**1a–d**) were obtained by treating 2‐(6‐chloro‐9*H*‐carbazol‐2‐yl)propanoyl isothiocyanate (**2**) with aniline, *para*‐toluidine, *para*‐bromoaniline, and *para*‐iodoaniline, respectively. The isothiocyanate **2** was obtained in situ by refluxing 2‐(6‐chloro‐9*H*‐carbazol‐2‐yl)propanoyl chloride (**3**) with ammonium thiocyanate using anhydrous acetone as the reaction medium. The acid chloride **3** was synthesized by treating carprofen (**4**) with excess thionyl chloride.

The synthesis route for the obtaining of the target compounds is shown in Scheme 1.

**SCHEME 1:**
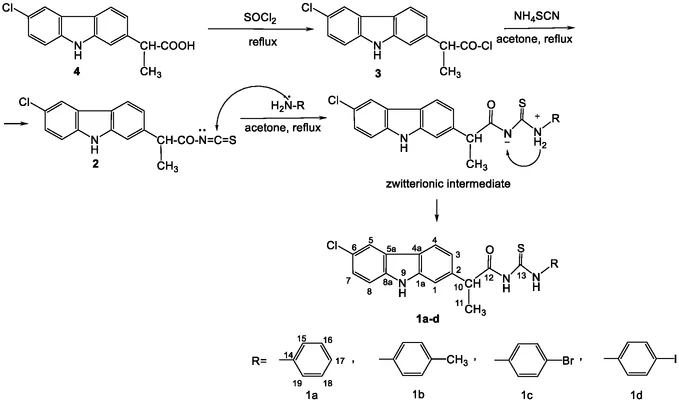
Synthesis pathway for the novel compounds.

The reaction of acyl isothiocyanate (2) with the amine proceeds via a nucleophilic addition pathway. In the initial step, the amine nitrogen acts as a nucleophile, attacking the electrophilic carbon atom of the isothiocyanate group. This leads to the formation of a transient zwitterionic intermediate, which subsequently undergoes rapid proton transfer to afford the corresponding *N*‐acylthiourea derivative [51].

In order to obtain the compounds with high purity and the best possible yields, we established the optimal reaction conditions.

The structures of the original compounds were confirmed using Fourier transform infrared spectroscopy (FT‐IR), nuclear magnetic resonance spectroscopy (^1^H‐NMR, ^13^C‐NMR), and high‐resolution mass spectrometry (HRMS). For the correct assignment of the NMR signals, complementary 2D‐NMR spectra were performed.

The novel derivatives **1a** **–d** were characterized by their physical constants (melting point, solubility).

The results obtained using spectral analysis and mass spectra confirm both the structure of the novel compounds and the performed syntheses.

In the IR spectra, the N—H stretching vibrations of the amide group appeared at 3337–3398 cm^−1^. The antisymmetric methyl C—H stretching vibrations were observed at 2965–2972 cm^−1^. The derivatives were characterized by absorptions at 1677–1681 cm^−1^ for the carbonyl group, lower than the usual carbonyl absorption (1730 cm^−1^), due to intramolecular hydrogen bonding between the carbonyl oxygen and a nitrogen‐bound hydrogen atom. A very strong absorption characteristic of amide N—H was observed at 1519–1529 cm^−1^. The band situated at 1339–1344 cm^−1^ was attributed to the thioamide group, while the signals at 1256–1259 cm^−1^ were assigned to C=S stretching bands.

In the ^1^H‐NMR spectra, the proton of the CS–NH–Ar group was observed at 12.39–12.46 ppm and that of the CO–NH–CS group at 11.43–11.44 ppm, both as singlets. H‐9 also appears as a singlet, at 11.64–11.73 ppm. H‐10 appears as a quartet at a chemical shift of approximately 4.24–4.25 ppm, and H‐11 exhibits a doublet multiplicity at a chemical shift value around 1.50–1.51 ppm. The methyl group protons of the compound **1b** are found as a singlet at 2.28 ppm.

In the ^13^C‐NMR, the characteristic signals were assigned to the C=S and C=O carbons at *δ* 178.79–179.19 and 176.05–176.20 ppm, respectively. Also, signals for C‐10 and C‐11 appear in the regions of 45.42–45.50 and 18.30–18.38 ppm.

For all compounds, APCI+ high‐resolution mass spectra were recorded in MeCN. The [M+H]^+^ molecular peaks were observed as base peaks for three compounds (**1a**, **1b**, and **1d**), while for **1c**, the molecular peak was identified with a relative intensity of 76%. The base peak at m/z 273.07936 in the mass spectrum of **1c** corresponds to the [C_15_H_14_ClN_2_O]^+^ fragment, whose structure is shown in Figure 1. Another peak observed in the mass spectra of all compounds, with relative intensities in the range of 58%–67%, corresponds to the [C_14_H_11_ClN]^+^ cation at a calculated m/z value of 228.05800. For all studied compounds, the peak corresponding to the [M‐S‐H]^+^ fragment was also observed, with relative intensities between 5% and 15%.

**FIGURE 1:**
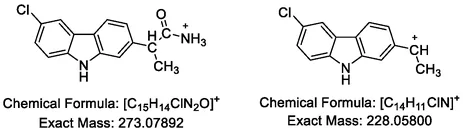
The structure of [C_15_H_14_ClN_2_O]^+^ and [C_14_H_11_ClN]^+^ fragments identified in the APCI‐MS spectra.

### 2.2 Antimicrobial Activity

Table 1 presents the quantitative parameters of antimicrobial activity.

**TABLE 1:** Quantitative evaluation of antimicrobial activity expressed as minimum inhibitory concentration (MIC), minimum microbicidal concentration (MMC), and minimum biofilm eradication concentration (MBEC).

Compounds		*Staphylococcus aureus* ATCC 25923	*Enterococcus faecalis* ATCC 19433	*Pseudomonas aeruginosa* ATCC 27853	*Klebsiella pneumoniae* ATCC 13368	*Candida albicans* ATCC 10231
**1a**	MIC, mg/mL	0.50	0.31	1.25	1.25	0.625
	MMC, mg/mL	5	>5	2.5	2.5	2.5
	MBEC, mg/mL	0.625	0.625	1.25	1.25	0.625
**1b**	MIC, mg/mL	0.25	2.5	1.25	1.25	1.25
	MMC, mg/mL	5	>5	2.5	5	5
	MBEC, mg/mL	0.125	0.625	1.25	1.25	0.625
**1c**	MIC, mg/mL	5	0.31	1.25	1.25	0.625
	MMC, mg/mL	5	>5	2.5	2.5	2.5
	MBEC, mg/mL	0.625	0.625	1.25	1.25	0.16
**1d**	MIC, mg/mL	5	1.25	1.25	>5	0.16
	MMC, mg/mL	>5	>5	2.5	2.5	2.5
	MBEC, mg/mL	1.25	1.25	1.25	1.25	0.16
Gentamicin	MIC, µg/mL	4.38	140	8.75	4.38	—
	MMC, µg/mL	17.5	140	8.75	70	—
	MBEC, µg/mL	4.38	140	8.75	4.38	—
Ketoconazole	MIC, µg/mL	—	—	—	—	17.5
	MMC, µg/mL	—	—	—	—	7
	MBEC, µg/mL	—	—	—	—	17.5
DMSO	MIC, mg/mL	2.5	2.5	1.25	2.5	1.25
	MMC, mg/mL	>5	>5	2.5	2.5	5
	MBEC, mg/mL	1.25	2.5	1.25	1.25	1.25

In quantitative assays, the compounds demonstrated varying antibacterial activity, with minimum inhibitory concentration (MIC) values ranging from 0.16 to 5 mg/mL, depending on the chemical structure of compounds and the microbial strain tested.

The most effective compound against *Staphylococcus aureus* strains was compound **1b** (MIC = 0.25 mg/mL), and against *Enterococcus faecalis* were **1a** and **1c** (MIC = 0.31 mg/mL). None of the compounds had any effect against the *Pseudomonas aeruginosa* strain. In the case of the *Klebsiella pneumoniae* strain, compounds **1a**, **1b**, and **1c** have lower MIC values (1.25 mg/mL) compared to DMSO (2.5 mg/mL). The active compounds against the yeast strain of *Candida albicans* with a MIC value lower than the corresponding DMSO concentration were **1a**, **1c**, and **1d**, the last one being the most active (MIC = 0.16 mg/mL). In Table 1, MIC values lower than those of the solvent were highlighted in orange.

In the case of the *S. aureus* strain, a more pronounced microbicidal effect was observed compared to that of the solvent for compounds **1a**, **1b**, and **1c** (MIC = 5 mg/mL), while for *C. albicans*, the most active compounds were **1a**, **1c**, and **1d** (highlighted in green in Table 1).

In the case of the Gram‐negative strains and the Gram‐positive strain, *E. faecalis*, none of the compounds showed a microbicidal effect at concentrations lower than the used solvent. Therefore, the microbicidal effect was generated by the solvent. The representative images presented in Figure S21 illustrate the determination of the minimum microbicidal concentration (MMC) of the tested compounds, highlighting the absence of microbial growth at the concentrations with a microbicidal effect.

By statistically analyzing the IC50 values, it was observed that some of the compounds had significantly higher antimicrobial activity compared to the solvent used (DMSO, *p* < 0.05). The IC50 values were expressed both in mg/mL and in mM, simultaneously, for the gravimetric and molecular evaluation of antimicrobial activity. The expression in mg/mL was maintained to allow correlation with the solvent control, while the molar expression permitted comparison of the intrinsic activity of structurally different compounds. In the case of the *S. aureus* strain (Figure 2), antimicrobial activity was observed in the following order: gentamicin > **1b** > **1a** > DMSO > **1d** > **1c**. The reduced activity of the halogenated derivatives may be associated with steric effects or altered electronic properties induced by the larger bromine and iodine atoms. The antimicrobial activity against *S. aureus* seems to be correlated with the presence of the methyl group in the *para* position, thus slightly increasing the molecule's lipophilicity, while bulky substituents tend to reduce activity [52].

The efficacy against *E. faecalis* (Figure 2) was in the following order: **1a** < **1c** < **1d** < **1b** < DMSO. Small molecular volume and the absence of large halogen substituents were associated with increased antimicrobial activity. The IC50 values expressed in mM revealed that compound **1b**, bearing the methyl group, showed the lowest antimicrobial potency against *E. faecalis*, followed by the iodine‐substituted derivative **1d**. Halogenated substituents (Br, I) can reduce activity, possibly by interfering with membrane permeability or target binding [53, 54].

**FIGURE 2:**
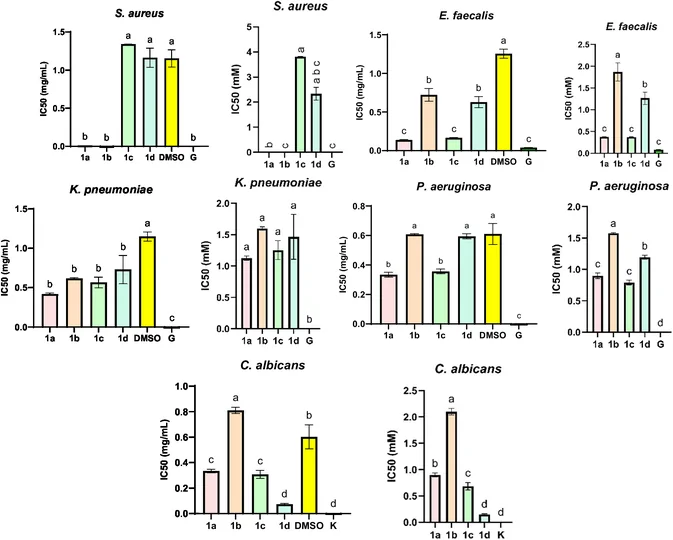
Antimicrobial activity expressed as IC50 values in mg/mL (gravimetric perspective) and mM (molecular perspective). Correlation with the activity of the solvent control was assessed only for IC50 values expressed in mg/mL. Distinct letters (a–d) above the bars indicate significant differences between samples (*p* < 0.05). Data represent the mean of two independent experiments (*n* = 2), and error bars indicate the standard deviation of the means.

In the case of the Gram‐negative *K. pneumoniae* strain (Figure 2), the studied compounds had significantly lower IC50 values compared to DMSO (*p* < 0.05), but significantly higher compared to the antibiotic used as a positive control (gentamicin) (*p* < 0.05). The differences between the tested compounds were not significant. The IC50 values expressed in mM indicated comparable antimicrobial activity of the tested compounds against *K. pneumoniae*, although the iodine‐substituted derivative **1b** showed a tendency toward lower potency.

For the *P. aeruginosa* strain (Figure 2), the order of antimicrobial activity was as follows: **1a** > **1c** > **1d** > **1b** > DMSO. In contrast to compounds **1a** and **1c**, which showed substantially lower IC50 values (*p* < 0.05), compounds **1b** and **1d** did not show a significant difference from DMSO. The molecule with the strongest antibacterial activity, **1a**, had a simpler and sterically unrestricted structure, indicating better cellular permeability and efficient contact with bacterial targets. The molar expression of IC50 values highlighted the influence of structural substitutions on antimicrobial activity against *P. aeruginosa*. The methyl‐substituted derivative **1b** and the iodine‐containing compound **1d** displayed reduced intrinsic potency, while bromine substitution in compound **1c** appeared to preserve antimicrobial activity. Only a methyl group separates **1b** from **1a**; however, this alteration seems to drastically lower activity, perhaps by changing the steric or electrical characteristics. Compound **1d**, which includes iodine, exhibited moderate activity, most likely as a result of its increased volume and effect on Gram‐negative bacterial permeability through the outer membrane.

In the case of the *C. albicans* strain, the differences were significant (Figure 3) compared to the solvent, with compound **1d** having similar values to the standard antifungal (ketoconazole) (*p* > 0.05), while compounds **1a** and **1c** have significantly higher IC50 values compared to **1d** and ketoconazole (*p* < 0.5), but significantly lower compared to the solvent. Compound **1d** exhibited superior antifungal activity compared to its antibacterial activity, suggesting a favorable structure–activity relationship for inhibiting *C. albicans*. The iodine substituent contributes to increased lipophilicity, which facilitates interaction with the ergosterol‐rich membranes of fungi. Unlike Gram‐negative bacteria, which have an additional barrier (the outer membrane), fungi allow for more efficient diffusion of large molecules. The molar expression of IC50 values demonstrated that replacing the methyl group with halogen substituents enhanced activity, especially for the iodine‐containing derivative **1d**, which showed the lowest IC50 value. Furthermore, the presence of iodine could induce oxidative stress or directly affect fungal membrane structures, which may explain the significant difference in efficacy [55].

It was also evaluated the influence of the newly synthesized compounds on microbial adherence which represents the initial stage of microbial biofilm development. The minimum biofilm eradication concentration (MBEC) values are presented in Table 1. Compounds with MBEC values lower than the MIC values and MBEC for DMSO were considered effective in inhibiting microbial adherence (these values are highlighted in blue in Table 1). For the *S. aureus* strain, only compounds **1b** and **1c** inhibited adherence, while the microbial adherence of *E. faecalis* cells was inhibited only by compound **1b**. The adherence of Gram‐negative strains of *K. pneumoniae* and *P. aeruginosa* was not inhibited by any compound. Only compounds **1b** and **1c** inhibited the adherence of *C. albicans* cells at MBEC values lower than the MIC (blue marking in Table 1). The representative micrographs presented in Figure S22 highlight the decrease in microbial cells’ adherence to the inert substrate after exposure to MBEC and MBEC/2 concentrations, compared to untreated controls, suggesting an inhibitory effect on the adhesion process. Although several microbial strains were investigated, the figure exclusively presents the isolates of *E. faecalis*, *K. pneumoniae*, and *C. albicans*, selected as representative examples, where the antibiofilm activity of the tested compound was most clearly demonstrated.

### 2.3 Antioxidant Activity

The antioxidant activity of the four analyzed compounds (**1a–d**) was evaluated using four complementary methods: IC50 DPPH (2,2‐diphenyl‐1‐picrylhydrazyl radical), CUPRAC (CUPric Reducing Antioxidant Capacity), FRAP (Ferric Reducing Antioxidant Power), and ABTS (2,2′‐azino‐bis‐(3‐ethylbenzothiazoline‐6‐sulfonic acid)).

Compound **1a** demonstrated the best overall antioxidant activity and exhibited the lowest IC50 value (Figure 3a), indicating high efficiency in neutralizing DPPH radicals, as well as the highest activity in the FRAP (Figure 3c) and ABTS (Figure 3d) assays. However, in the CUPRAC test (Figure 3b), its activity was relatively lower, suggesting a weaker interaction with copper ions.

Compound **1b** showed a moderate antioxidant profile in all tests. The extended aromatic system contributed to a moderate electron‐donating capacity, which resulted in good performance in FRAP (Figure 3c) and ABTS (Figure 3d) tests and reasonable performance in CUPRAC (Figure 3b).

Compound **1c** showed reduced antioxidant activity, particularly in the DPPH (Figure 3a) and FRAP (Figure 3c) tests, suggesting a weak radical scavenging capacity. The presence of the bromine atom on the aromatic ring may be responsible for this behavior, through electron‐withdrawing inductive effects and potential steric interference.

Compound **1d** recorded the lowest values in all tests, especially in FRAP (Figure 3c) and ABTS (Figure 3d). The iodine atom, being larger and a stronger electron acceptor, appears to have a negative impact on antioxidant capacity, possibly by reducing the electron density available for interaction with free radicals.

**FIGURE 3:**
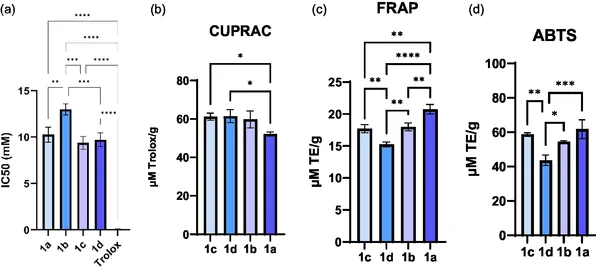
Comparative antioxidant profile of compounds **1a–d** determined by four spectrophotometric methods: (a) DPPH, (b) CUPRAC, (c) FRAP, and (d) ABTS/TEAC.

### 2.4 Hemocompatibility and Antihemolytic Activity

A correlation study of the antimicrobial activity and hemolytic profile of newly synthesized compounds showed that enhanced activity does not always reflect increased cytotoxicity (Figure 4a). With a low hemolysis percentage, compounds **1c** and **1d** provide the best possible balance between safety and effectiveness, especially for *C. albicans* and Gram‐positive bacteria strains. The most hemolytic, however, was **1b**, which suggests a poor pharmacological profile while being comparatively microbiologically active. These findings suggest that optimizing chemical structures for selectivity against bacteria while avoiding human cells is critical in the development of antimicrobial drugs. ISO 10993‐4:2017 [56] defines hemocompatibility as a substance that causes less than 5% hemolysis. In this context, compounds **1c** and **1d** meet this criterion, indicating a favorable safety profile for potential therapeutic uses.

**FIGURE 4:**
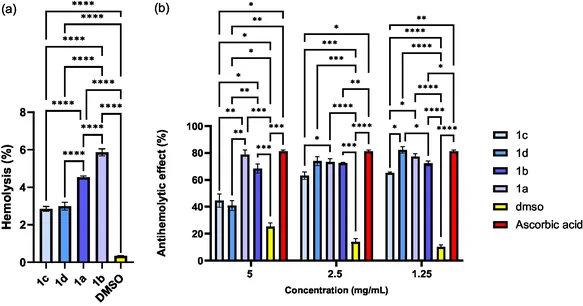
Antihemolytic activity of newly synthesized compounds: (a) hemolysis (%) induced by the tested compounds at 5 mg/mL and (b) antihemolytic activity against AAPH‐induced oxidative hemolysis in sheep erythrocytes (*p < 0.05, **p < 0.01, ***p < 0.001, ****p < 0.0001).

Analysis of the antihemolytic effect (Figure 4b) on sheep erythrocytes subjected to oxidative stress induced by AAPH revealed that only **1a** and **1b** provide consistent and significant protection across the entire range of concentrations tested (5–1.25 mg/mL). In contrast, halogenated compounds **1c** and **1d** showed a relevant protective effect only from 2.5 mg/mL, indicating dose‐dependent efficacy. This trend was also supported by the results of classical antioxidant tests (DPPH, FRAP, and ABTS), where **1a** and **1b** showed superior antioxidant capacity. Specifically, **1a** had the lowest IC50 in the DPPH assay and the best results in FRAP and ABTS, indicating excellent free radical scavenging capacity. Data correlation suggests that **1a** and **1b** possess a dual advantage, namely, high chemical antioxidant capacity and biological efficacy in protecting erythrocyte membranes, even at low doses. In contrast, **1c** and **1d**, although active at higher doses, exhibit limited antioxidant efficacy under biological conditions, despite moderate chemical activity.

### 2.5 Cytotoxicity Assessment

Cell viability was assessed using the CellTiter Aqueous One Solution Cell Proliferation Assay, a colorimetric method based on the bioreduction of the tetrazolium compound MTS [3‐(4,5‐dimethylthiazol‐2‐yl)‐5‐(3‐carboxymethoxyphenyl)‐2‐(4‐sulfophenyl)‐2*H*‐tetrazolium] by metabolically active cells into a soluble formazan product that is produced in direct proportion to the number of viable cells and their metabolic activity. As shown in Figure 5, all four compounds induced a dose‐dependent decrease in the viability of all four cell lines. At low concentrations (7.85–42.00 µM), cell viability remained close to control levels (approximately 90%–100%), indicating limited cytotoxic activity. A‐431 epidermoid carcinoma cells were the most sensitive, particularly to compounds **1b** (IC50 = 35.42 µM) and **1a** (IC50 = 42.04 µM) (Table 2). This suggests increased sensitivity of A‐431 cells to these structures. Cytotoxicity was moderate in MG‐63 cells (IC50 between 58.56 and 74.17 µM), with close values for **1a** **–c** suggesting a comparable effect and no major differences in potency among these derivatives. In HT‐29 and HCT‐8 cells, relatively high IC50 values (61.11–111.57 µM) indicated greater resistance. Compounds **1a** **–c** displayed similar moderate activity (approximately 58.56–74.17 µM), whereas **1d** showed low activity on HT‐29 cells (IC50 = 111.57 µg/mL), being the weakest derivative in this model. The observed pattern indicates possible selective cytotoxicity toward A‐431 cells and suggests a structure–activity relationship, with compound **1b** being the most promising candidate for further studies.

**FIGURE 5:**
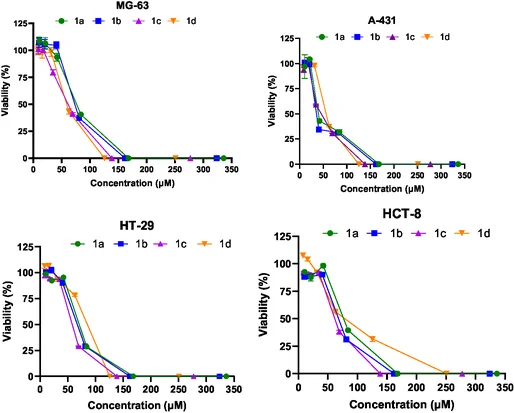
Dose‐response curves of compounds **1a** **–d** on MG‐63, A‐431, HT‐29, and HCT‐8 cell lines following 72 h of treatment, assessed by the MTS assay.

**TABLE 2:** Comparative IC50 values (µM) of compounds **1a** **– d** in MG‐63, A‐431, HT‐29, and HCT‐8 cell lines, determined using the MTS assay after 72 h of treatment. Lower IC50 values indicate higher cytotoxic potency.

Compound	MG‐63	A‐431	HT‐29	HCT‐8
**1a**	72.26	42.04	78.28	82.58
**1b**	74.17	35.42	65.98	78.13
**1c**	58.56	42.44	61.11	67.87
**1d**	61.47	57.87	111.57	66.47

### 2.6 Molecular Docking Study

Molecular docking analysis of the 3TTZ structure [57] yielded binding affinities ranging from −9.1 to −7.7 kcal/mol, with compound **1a** exhibiting the most favorable binding score. Furthermore, docking simulations against the 4EEQ structure resulted in even stronger binding affinities, ranging from −10.9 to −9.6 kcal/mol. The most favorable binding scores for both protein targets are summarized in Table 3.

**TABLE 3:** Names of the evaluated ligands, their minimized energies using the universal force field (*uff*), and binding affinities obtained from molecular docking and interacting amino acids.

Ligand	Binding affinity	Interacting amino acids
3TTZ_1a_*uff*_E = 650.14	−9.1	SER55, ILE51, GLY83, ASP81, LEU103, ILE102, THR173, ILE175, ASN54, ILE86, GLU58, GLY85, PRO87, ARG84, ARG144
3TTZ_1b_*uff*_E = 685.53	−8.5	ILE51, ILE86, LEU103, ASP81, SER55, ASN54, GLU58, PRO87, ASP57, ILE102, GLY85, THR173, ILE175
3TTZ_1c_*uff*_E = 606.41	−8.5	ILE51, ILE175, LEU103, ILE86, ASP81, SER55, ASN54, PRO87, GLU58, ARG84, GLY85, THR173
3TTZ_1d_*uff*_E = 564.05	−8.7	LEU205, TYR141, LYS78, THR171, GLY172, LYS170, ASN82, ASP81, THR80, GLN66, GLU68, ILE67, GLN210, THR212, HIS143, VAL174
4EEQ_1a_*uff*_E = 650.14	−10.7	GLU118, LEU119, ILE121, VAL289, SER88, ARG173, THR228, MET86, TYR87, PRO85, ILE84, PHE303, GLN298, TRP310, ALA125, LYS291, **TYR227**, LEU89, LYS120
4EEQ_1b_*uff*_E = 685.53	−10.8	PHE303, TRP310, MET86, TYR87, PRO85, ALA125, TYR227, LYS291, LYS120, LEU119, ILE121, GLU118, VAL289, LEU89, SER88, THR228
4EEQ_1c_*uff*_E = 606.41	−10.5	PRO85, TYR87, LEU89, LYS120, VAL289, **TYR227**
4EEQ_1d_*uff*_E = 564.05	−10.9	ASP299, PHe295, PHE232, GLN298, PHE303, TRP310, THR228, PRO308, TYR87, VAL289, LEU89, LYS120, GLU175, ILE121, TYR227, LYS291, GLU118, ARG173, ALA230

Molecular docking analysis of the ligands within the active site of the target macromolecule, DNA gyrase (PDB ID: 3TTZ, a type II topoisomerase), revealed distinct binding modes, as illustrated in Figure 6. Notably, compounds **1a**, **1b**, and **1c** exhibited a highly conserved binding pose, sharing a core network of eleven interacting residues: ASN54, ILE51, SER55, GLU58, ASP81, ILE86, GLY85, PRO87, LEU103, THR173, and ILE175. This extensive overlap strongly suggests that these three compounds occupy the same binding pocket. In contrast, compound **1d** adopted a markedly different binding orientation and did not share this conserved interaction network. The critical role of specific residues in this pocket is well‐documented; for instance, Sherer et al. emphasized the importance of ASP81, ARG84, and ARG144 in mediating ligand interactions within the active site of DNA gyrase. Intriguingly, compound **1a** was the only ligand predicted to interact with all three of these key residues. Furthermore, the interaction with ASP81 was conserved across the complexes of compounds **1b**, **1c**, and **1d**. Given the essential role of this residue in the enzyme's catalytic mechanism, the ability of these ligands to anchor to ASP81 may be a key structural determinant contributing to their enhanced antibacterial spectrum [57].

**FIGURE 6:**
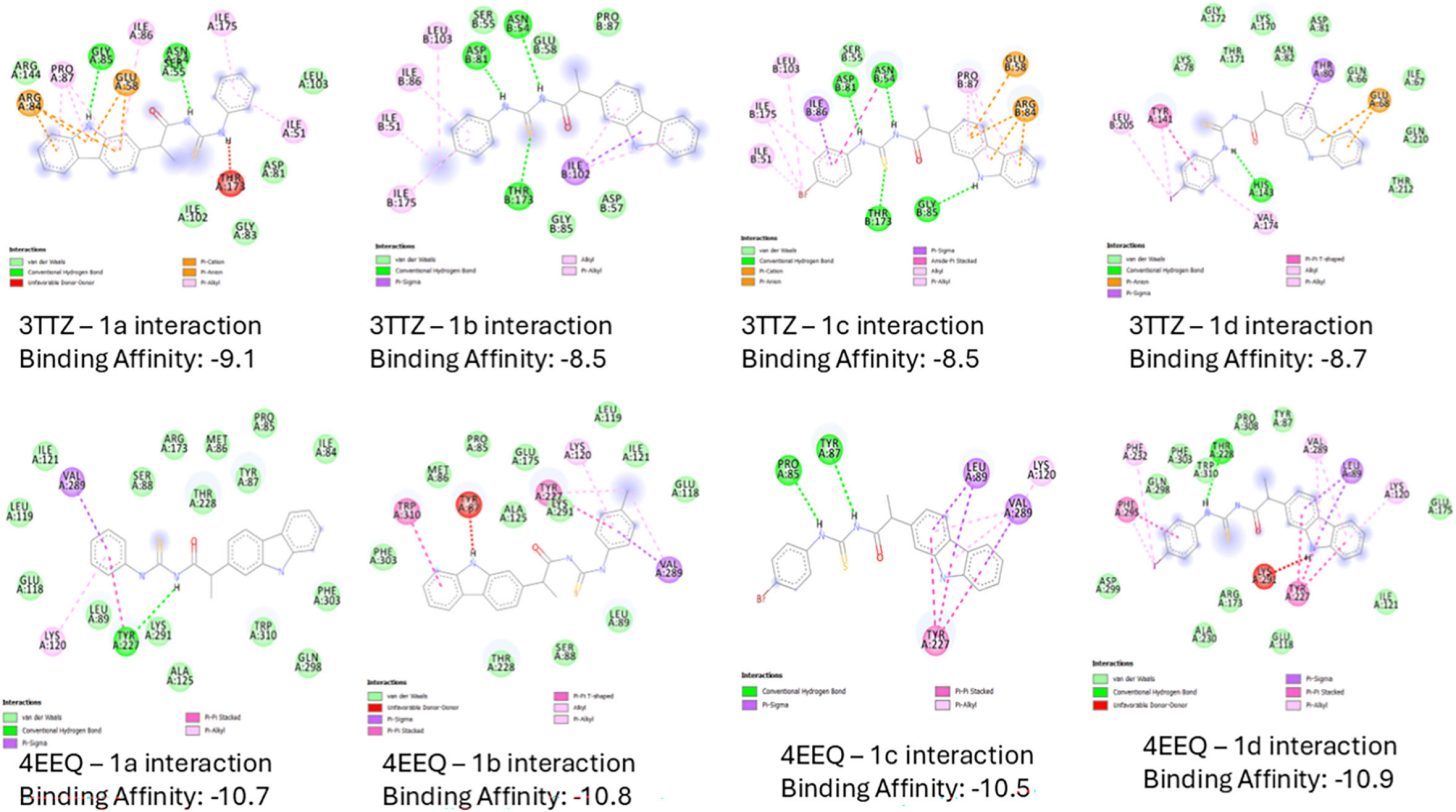
Binding affinity interaction for 3TTZ and 4EEQ structure with studied compounds.

Molecular docking analysis of the ligands within the active site of DNA ligase (PDB ID: 4EEQ) revealed a highly conserved binding mode among the evaluated compounds. Specifically, compounds **1a**, **1b**, **1c**, and **1d** share a universal core network consisting of five interacting residues (TYR87, LEU89, LYS120, VAL289, and TYR227), indicating that all four ligands occupy the same primary binding pocket. In addition, compounds **1a**, **1b**, and **1c** share an additional anchoring interaction with PRO85. In contrast, compounds **1a**, **1b**, and **1d** display a more extended binding pose, establishing secondary contacts with six other residues (GLU118, ILE121, THR228, LYS291, PHE303, and TRP310) that are absent in the complex with compound **1c**. The high degree of overlap in the primary binding interaction, in particular the conserved contacts with TYR87, LEU89, LYS120, VAL289, and TYR227, underscores the structural stability of these ligand–receptor complexes and highlights key residues that may be critical for mediating the observed biological activity [58].

### 2.7 In Silico Medicinal Chemistry, Pharmacokinetic, and Toxicity Profiling

All compounds exhibited favorable bioavailability and satisfied both Lipinski's rule of five and Veber's rule, reinforcing their drug‐like profiles and indicating strong potential for suitability in oral administration. These parameters collectively suggest that the compounds possess physicochemical characteristics commonly associated with successful orally active drugs. In the context of PAINS analysis, compounds **1a–d** could be included into a category of structures that may appear as “hits” during preliminary screening campaigns. According to the Brenk alert system, compounds **1a**–**c** each contain a thiocarbonyl group, a structural feature frequently flagged as potentially problematic due to its tendency to produce nonspecific biological effects. Compound **1d** includes two such alerting features, both a thiocarbonyl group and an iodine substituent, which may further increase the likelihood of assay interference. The assessment of the synthetic accessibility scores indicates that all four compounds are relatively easy to synthesize. This favorable accessibility enhances their practical value, potentially facilitating future development, optimization and scale‐up efforts.

All compounds are predicted to exhibit efficient intestinal absorption, supported by Caco‐2 permeability values ranging from –5.10 to –5.18. Regarding P‐glycoprotein interactions, **1c** and **1d** are inhibitors. All **1a** **–d** molecules demonstrate skin permeability, as reflected by LogKp values above –2.5. Moreover, all four compounds are anticipated to cross the blood–brain barrier (BBB) and penetrate the central nervous system (CNS), with logPS values between –1.49 and –1.28. Despite their moderate plasma protein binding (62%–69%), a substantial fraction of each compound remains unbound, enabling effective systemic exposure and distribution. None of the molecules are predicted to inhibit OATP1b3, although all inhibit OATP1b1 and OCT2 transporters. Finally, the predicted clearance values (2.47–2.82 mL/min/kg) indicate moderate elimination, while the short estimated half‐life (<3 h) suggests rapid systemic clearance (Table 4).

**TABLE 4:** Predicted pharmacokinetic profiles (absorption, distribution, and excretion) obtained using Deep‐PK (https://biosig.lab.uq.edu.au/deeppk/).

Properties	Compound				
	1a	1b	1c	1d	Unit
**Absorption**					
Caco‐2	−5.16	−5.10	−5.14	−5.18	logPaap, cm/s
Human intestinal absorption	Absorbed	Absorbed	Absorbed	Absorbed	Category (absorbed/nonabsorbed)
P‐Glycoprotein inhibitor	Noninhibitor	Noninhibitor	Inhibitor	Inhibitor	Category (inhibitor/noninhibitor)
P‐Glycoprotein substrate	Nonsubstrate	Nonsubstrate	Nonsubstrate	Nonsubstrate	Category (substrate/nonsubstrate)
Skin permeability	−1.71	−1.80	−1.56	−1.56	log Kp
**Distribution**					
Blood–brain barrier (central nervous system)	−1.49	−1.44	−1.35	−1.28	log PS
Blood–brain barrier (BBB)	Penetrable	Penetrable	Penetrable	Penetrable	Category (penetrating/nonpenetrating)
Plasma protein binding	62.36	68.57	65.61	64.34	Therapeutic index
**Excretion**					
OATP1b1	Inhibitor	Inhibitor	Inhibitor	Inhibitor	Category (inhibitor/noninhibitor)
OATP1b3	Noninhibitor	Noninhibitor	Noninhibitor	Noninhibitor	Category (inhibitor/noninhibitor)
Clearance	2.82	2.64	2.59	2.47	Log, mL/min/kg
Organic cation transporter 2 (OCT2)	Inhibitor	Inhibitor	Inhibitor	Inhibitor	Category (inhibitor/noninhibitor)
Half‐life of drug	Half‐life < 3 h	Half‐life < 3 h	Half‐life < 3 h	Half‐life < 3 h	Category (half‐life ≥ 3 h/half‐life < 3 h)

The in silico analysis showed that all compounds were predicted to inhibit CYP1A2, CYP2C19, CYP2C9, and CYP3A4, while acting as CYP2D6 substrates (Table 5). These findings are suggesting a potential risk for drug–drug interactions through interference with metabolic pathways and an interindividual variability in pharmacokinetics.

**TABLE 5:** Predicted pharmacogenomic profiles of compounds **1a–d** against major cytochrome P450 enzymes.

Properties	Compound				
	1a	1b	1c	1d	Unit
CYP 1a2 inhibitor	Inhibitor	Inhibitor	Inhibitor	Inhibitor	Category (inhibitor/noninhibitor)
CYP 1a2 substrate	Nonsubstrate	Substrate	Nonsubstrate	Nonsubstrate	Category (substrate/nonsubstrate)
CYP 2C19 inhibitor	Inhibitor	Inhibitor	Inhibitor	Inhibitor	Category (inhibitor/noninhibitor)
CYP 2C19 substrate	Nonsubstrate	Nonsubstrate	Nonsubstrate	Nonsubstrate	Category (substrate/nonsubstrate)
CYP 2C9 inhibitor	Inhibitor	Inhibitor	Inhibitor	Inhibitor	Category (inhibitor/noninhibitor)
CYP 2C9 substrate	Nonsubstrate	Nonsubstrate	Nonsubstrate	Nonsubstrate	Category (substrate/nonsubstrate)
CYP 2D6 inhibitor	Noninhibitor	Noninhibitor	Noninhibitor	Noninhibitor	Category (inhibitor/noninhibitor)
CYP 2D6 substrate	Substrate	Substrate	Substrate	Substrate	Category (substrate/nonsubstrate)
CYP 3A4 inhibitor	Inhibitor	Inhibitor	Inhibitor	Inhibitor	Category (inhibitor/noninhibitor)
CYP 3A4 substrate	Nonsubstrate	Nonsubstrate	Nonsubstrate	Substrate	Category (substrate/nonsubstrate)

A broad toxicity panel was considered, including (i) organ toxicity: hepatotoxicity, neurotoxicity, nephrotoxicity, respiratory toxicity and cardiotoxicity; (ii) toxicity endpoints: carcinogenicity, immunotoxicity, mutagenicity, cytotoxicity, and BBB toxicity; (iii) Tox21 nuclear receptor signaling pathways: aryl hydrocarbon receptor (AhR), androgen receptor (AR), androgen receptor ligand‐binding domain (AR‐LBD), aromatase, estrogen receptor ligand‐binding domain (ER‐LBD), and androgen receptor; and (iv) Tox21 stress response pathways: peroxisome proliferator‐activated receptor gamma (PPAR‐gamma), nuclear factor erythroid 2‐related factor 2/antioxidant response element (nrf2/ARE), and heat shock factor response element (HSE). Table 6 presents the endpoints for which compounds **1a** **–d** recorded a significant prediction probability (higher than 0.70).

**TABLE 6:** Toxicity prediction analysis performed with ProTox 3.0 (https://tox.charite.de/protox3/).

Toxicity prediction and probability	Compound			
	1a	1b	1c	1d
**Organ toxicity/probability**				
Cardiotoxicity	Inactive/0.85	Inactive/0.86	Inactive/0.85	Inactive/0.85
**Toxicity end points/probability**				
Immunotoxicity	Inactive/0.98	Inactive/0.98	Inactive/0.90	Inactive/0.96
Cytotoxicity	Inactive/0.73	Inactive/0.74	Inactive/0.62	Inactive/0.69
BBB	Active/0.75	Active/0.75	Active/0.74	Active/0.74
**Tox21 nuclear receptor signaling pathways/probability**				
Aryl hydrocarbon receptor (AhR)	Active/0.70	Active/0.69	Active/0.71	Active/0.70
Androgen receptor (AR)	Inactive/0.89	Inactive/0.89	Inactive/0.92	Inactive/0.92
Androgen receptor ligand binding domain (AR‐LBD)	Inactive/0.88	Inactive/0.89	Inactive/0.90	Inactive/0.90
Aromatase	Inactive/0.81	Inactive/0.79	Inactive/0.83	Inactive/0.84
Estrogen receptor ligand binding domain (ER‐LBD)	Inactive/0.93	Inactive/0.94	Inactive/0.93	Inactive/0.94
**Tox21 stress response pathways**				
Peroxisome proliferator activated receptor gamma (PPAR‐Gamma)	Inactive/0.95	Inactive/0.95	Inactive/0.96	Inactive/0.96
Nuclear factor (erythroid‐derived 2)‐like 2/antioxidant responsive element (nrf2/ARE)	Inactive/0.91	Inactive/0.90	Inactive/0.91	Inactive/0.91
Heat shock factor response element (HSE)	Inactive/0.91	Inactive/0.90	Inactive/0.91	Inactive/0.91

According to ProTox 3.0 predictions, the compounds exhibit an overall favorable safety profile, although they show a moderate probability of aryl hydrocarbon receptor (AhR) activation (0.70–0.71). Compound **1d** displayed an increased probability of hepatotoxicity (0.70) and all compounds were predicted to show BBB activity (0.74–0.75).

### 2.8 Integrated Biological Profile and Structure–Activity Considerations

The results confirm the antimicrobial potential of *N*‐(arylcarbamothioyl)propanamide derivatives.

The strains chosen for testing have high clinical relevance and the ability to develop resistance to current treatments.

The compounds showed good antimicrobial activity against *S. aureus* and *E. faecalis*, indicating increased efficiency against Gram‐positive bacteria. This selectivity could be explained by differences in cell wall composition, which allow easier penetration of molecules through the cell wall of Gram‐positive bacteria.

Certain structural features can improve antimicrobial specificity and efficacy. Compound **1b** was the most active against *S. aureus* strain and compounds **1a** and **1c** against *E. faecalis* strain. The activity was lower against Gram‐negative bacteria. Compounds **1a**, **1b**, and **1c** were the most active against *K. pneumoniae*. Compound **1d** proved to be the most active against *C. albicans*. Compounds **1b** and **1c** inhibited *S. aureus* microbial adherence and compound **1b** inhibited *E. faecalis* microbial adherence. Compound **1c** was the most potent inhibitor of microbial adherence of the *C. albicans* strain with an MBEC value = 0.16 mg/mL.

Antioxidant activity was determined by free radical neutralization capacity (using DPPH assay), by the reduction capacity of Fe^3+^ to Fe^2+^ (FRAP assay), the reduction capacity of Cu^2+^ to Cu^+^ (CUPRAC assay), and total antioxidant capacity against ABTS^+^• radical (TEAC assay). Compound **1a** showed the most promising antioxidant potential, followed by compound **1b**. The presence of halogens (Br and I) in the structure of compounds **1c** and **1d**, respectively, significantly reduces their antioxidant activity, both through steric and electronegative effects. This observation highlights the importance of choosing appropriate substituents in the design of molecules with antioxidant potential.

The highlighted antioxidant capacity supports the use of these compounds in potential treatments involving oxidative stress, including in chronic infections where inflammation plays an important role.

Compound **1a** exhibited both good antimicrobial activity and significant antioxidant activity, suggesting a possible synergistic effect. This dual property is particularly valuable in combating infections associated with oxidative stress.

An essential aspect for obtaining safe and effective compounds against microorganisms is also related to safety for human cells, a hemolytic profile that includes minimal hemolysis, at the same compound concentrations.

Compounds **1a** and **1b** possess a dual advantage, namely, biological efficacy in protecting erythrocyte membranes, even at low doses and high chemical antioxidant capacity. In contrast, halogenated analogs **1c** and **1d** provided a relevant antihemolytic protective effect only at a concentration of 2.5 mg/mL, thus demonstrating a dose‐dependent efficacy.

In our evaluation, we observed variable cytotoxic activity across the analyzed cell lines. The derivatives **1a**–**1c** showed moderate potency against MG‐63 and colon cancer cell lines, while compound **1b** demonstrated the highest overall activity, particularly against A‐431 cells (IC50 = 13.67 µg/mL). In contrast, compound **1d** displayed the weakest effect, especially on HT‐29 cells (IC50 = 55.56 µg/mL).

Structure–activity relationship analysis revealed that biological activity is influenced by the nature of the *para*‐substituent attached to the phenyl ring. The *para*‐methyl derivative **1b** exhibited the best overall antimicrobial, antibiofilm, and cytotoxic profile, suggesting that moderate lipophilicity is beneficial for biological activity. In contrast, halogen substitution, especially iodine, generally reduced antibacterial and antioxidant properties but enhanced antifungal activity against *C. albicans*. The unsubstituted analog **1a** showed the strongest antioxidant and antihemolytic effects, indicating that electron‐donating environments are favorable for radical scavenging activity. These findings demonstrate that subtle electronic and steric modifications significantly affect the biological performance of carbazole–acylthiourea derivatives.

These results suggest a potential structure–activity relationship, with compound **1b** emerging as the most promising candidate for further mechanistic and apoptotic pathway investigations.

## 3 Experimental Section

### 3.1 General Information

Chemicals and solvents were purchased from Merck, Aldrich (Darmstadt and Steinheim, Germany), Fluka (Buchs, Switzerland), Chimopar, and Chimreactiv S.R.L. (Bucharest, Romania). Acetone was dried over calcium chloride and then distilled at normal pressure; ammonium thiocyanate was dried by heating at 100 °C.

### 3.2 Measurements

Melting points were determined using an Electrothermal 9100 apparatus (Bibby Scientific Ltd., Stone, United Kingdom) in open capillary tubes and were uncorrected.

IR spectra were recorded on a Bruker Vertex 70 FT‐IR spectrometer using the ATR technique (Bruker Corporation, Billerica, MA, United States). Frequencies were expressed in cm^−1^, with the following abbreviations: w = weak, m = medium, s = strong, vs = very strong, and sh = shoulder (Figures S1, S6, S11, and S16).

^1^H‐NMR and ^13^C‐NMR spectra were recorded using a Bruker Avance II 500 MHz spectrometer; chemical shifts are given in ppm relative to TMS (tetramethylsilane) as an internal standard.

In the ^1^H‐NMR spectra, chemical shifts were recorded as *δ* values, in parts per million (ppm) and coupling constants (*J*) in Hertz. Standard abbreviations indicating the multiplicity of signals are used as follows: s (singlet), d (doublet), q (quartet), m (multiplet), dd (doublet doublet), bs (broad singlet), and bd (broad doublet). ^1^H‐NMR data are reported in the following order: chemical shifts, multiplicity, number of protons and signal/atom assignment, and coupling constants (Figures S2, S7, S12, and S17).

For ^13^C‐NMR data, the order is as follows: chemical shifts and signal/atom assignment (Figures S3, S8, S13, and S18).

The APCI+ high‐resolution mass spectra for compounds **1a–d** were recorded on a Thermo Scientific LTQ‐Orbitrap XL spectrometer equipped with a standard ESI/APCI source. Thermo Xcalibur software was used for the processing of the mass spectra (Xcalibur Software, Thermo Fisher Scientific, Waltham, MA, United States, www.thermofisher.com) (Figures S4, S5, S9, S10, S14, S15, S19, and S20).

### 3.3 Chemical Synthesis and Spectral Characterization

#### 3.3.1 *Synthesis of 2‐*(*6‐Chloro‐9H‐carbazol‐2‐yl*)*propionic Acid Chloride*

In a round‐bottomed flask equipped with an ascending condenser and a calcium chloride tube, 0.02 mol of carprofen (MW 273.72) was introduced and dissolved in thionyl chloride (MW 118.97; d_4_^20^ 1.638). The mixture was refluxed for 3 h. The excess thionyl chloride was removed by distillation under reduced pressure. For the next step, 2‐(6‐chloro‐9*H*‐carbazol‐2‐yl)propionic acid chloride was used in crude form.

#### 3.3.2 *General Procedure for the Synthesis of New Carbazole–Acylthiourea Propanamides* (*1a–d*)

A solution of 0.01 mol of 2‐(6‐chloro‐9*H*‐carbazol‐2‐yl)propionic acid chloride (MW 292.156) in 15 mL of anhydrous acetone was added to a solution of 0.01 mol of ammonium thiocyanate (MW 76.13) in 5 mL of anhydrous acetone. The reaction mixture was refluxed for 1 h in a round‐bottomed flask fitted with a condenser and a calcium chloride tube. After cooling, 0.01 mol of primary aromatic amine dissolved in 2 mL of acetone was added to this reaction mixture under stirring. When working with liquid amine, this one was previously dried on potassium hydroxide and then distilled. The mixture was refluxed for 1 h. The crude product precipitated after the cooled reaction mixture was poured into 500 mL of water.

The obtained thiourea derivatives were crystallized from isopropanol–water (3:1), in the presence of activated charcoal.

#### 3.3.3 2‐(6‐Chloro‐9*H*‐carbazol‐2‐yl)‐*N*‐(phenylcarbamothioyl)propanamide (1a) (m.p. 145.4–148.7 °C; Yield 73%)

^**1**^ **H‐NMR** (500 MHz, dmso‐d6, *δ* ppm, *J* Hz): 12.46 (CS–HN–Ar); 11.67 (s, H‐9); 11.44 (CO–NH–CS); 8.18 (d, H‐5, 1.9); 8.13 (d, H‐4, 8.2); 7.60 (d, 2H, H‐15, H‐19, 7.6); 7.56 (bs, 1H, H‐1); 7.50 (d, 1H, H‐8, 8.6); 7.40÷7.36 (m, 4H, H‐7, H‐16, H‐17, H‐18); 7.23 (bd, 1H, H‐3, 8.2); 4.25 (q, H‐10, 7.0); 1.51 (d, H‐11, 7.0).

^**13**^ **C‐NMR** (125 MHz, dmso‐d6, *δ* ppm): 179.01 (C‐13); 176.20 (C‐12); 140.49 (C‐8a); 138.54 (C‐1a); 138.38 (C‐2); 137.68 (C‐14); 128.61 (C‐16, C‐18); 124.30 (C‐15, C‐19); 126.30 (C‐17); 125.26 (C‐7); 123.51 (C‐4a); 122.93 (C‐6); 120.79 (C‐5a); 120.78 (C‐4); 119.72 (C‐5); 118.61 (C‐3); 112.38 (C‐8); 110.17 (C‐1); 45.42 (C‐10); 18.30 (C‐11).

**FT‐IR** (ATR in solid, ν cm^−1^): 3337w; 3166m; 3030w; 2969w; 1677m; 1600w; 1525vs; 1460s; 1342m; 1258m; 1142s; 1064m; 1027sh; 932w; 865w; 800m; 692m.

Chemical formula C_22_H_18_ClN_3_OS, exact mass 407.08591, HRMS (APCI+, MeCN), m/z (%): 408.09254 (100) [M+H]^+^, 374.10498 (10) [M‐S‐H]^+^, 314.02711 (24) [C_16_H_11_ClN_2_OS]^+^, 273.07840 (57) [C_15_H_14_ClN_2_O]^+^, 255.04420 (17) [C_15_H_10_ClNO]^+^, 228.05699 (65) [C_14_H_11_ClN]^+^, 153.04840 (6) [C_7_H_9_N_2_S]^+^, 94.06507 (10) [C_6_H_8_N]^+^.

#### 3.3.4 2‐(6‐Chloro‐9*H*‐carbazol‐2‐yl)‐*N*‐(*para*‐tolylcarbamothioyl)propanamide (1b) (m.p. 175.5–178.7 °C; Yield 81%)

^**1**^ **H‐NMR** (500 MHz, dmso‐d6, *δ* ppm, *J* Hz): 12.39 (CS–HN–Ar); 11.64 (s, H‐9); 11.44 (CO–NH–CS); 8.19 (d, H‐5, 1.9); 8.13 (d, H‐4, 8.2); 7.55 (bs, 1H, H‐1); 7.50 (d, 1H, H‐8, 8.6); 7.47 (d, 2H, H‐15, H‐19, 8.0); 7.37 (dd, 1H, H‐7, 2.1, 8.6); 7.23 (bd, 1H, H‐3, 8.2); 7.17 (d, 2H, H‐16, H‐18, 8.0); 4.25 (q, H‐10, 7.0); 2.28 (s, 3H, CH_3_); 1.51 (d, H‐11, 7.0).

^**13**^ **C‐NMR** (125 MHz, dmso‐d6, *δ* ppm): 178.92 (C‐13); 176.20 (C‐12); 140.50 (C‐8a); 138.59 (C‐1a); 138.40 (C‐2); 135.66 (C‐17); 135.15 (C‐14); 129.06 (C‐16, C‐18); 125.27 (C‐7); 124.16 (C‐15, C‐19); 123.52 (C‐4a); 122.94 (C‐6); 120.79 (C‐4); 120.78 (C‐5a); 119.74 (C‐5); 118.59 (C‐3); 112.40 (C‐8); 110.18 (C‐1); 45.42 (C‐10); 20.56 (CH_3_); 18.31 (C‐11).

**FT‐IR** (ATR in solid, ν cm^−1^): 3398m; 3164w; 3030w; 2965w; 1679m; 1598m; 1519vs; 1462sh; 1344m; 1257m; 1136s; 1066m; 1023m; 871w; 805s; 726m; 614w.

Chemical formula C_23_H_20_ClN_3_OS, exact mass 421.10156, **HRMS** (APCI+, MeCN), m/z (%): 422.10937 (100) [M+H]^+^, 388.12165 (15) [M‐S‐H]^+^, 363.12647 (22), 315.03580 (15) [C_16_H_12_ClN_2_OS]^+^, 273.07937 (53) [C_15_H_14_ClN_2_O]^+^, 255.04497 (5) [C_15_H_10_ClNO]^+^, 228.05783 (58) [C_14_H_11_ClN]^+^, 214.08978 (6), 123.07907 (7).

#### 3.3.5 2‐(6‐Chloro‐9*H*‐carbazol‐2‐yl)‐*N*‐((4‐bromophenyl)carbamothioyl)propanamide (1c) (m.p. 191.9–195.6 °C; Yield 80%)

^**1**^ **H‐NMR** (500 MHz, dmso‐d6, *δ* ppm, *J* Hz): 12.41 (CS–HN–Ar); 11.73 (s, H‐9); 11.44 (CO–NH–CS); 8.18 (d, H‐5, 1.9); 8.13 (d, H‐4, 8.2); 7.59–7.54 (m, 4H, H‐15, H‐16, H‐18, H‐19); 7.55 (bs, 1H, H‐1); 7.50 (d, 1H, H‐8, 8.6); 7.37 (dd, 1H, H‐7, 2.1, 8.6); 7.23 (bd, 1H, H‐3, 8.2); 4.25 (q, H‐10, 7.0); 1.51 (d, H‐11, 7.0).

^**13**^ **C‐NMR** (125 MHz, dmso‐d6, *δ* ppm): 179.19 (C‐13); 176.12 (C‐12); 140.49 (C‐8a); 138.49 (C‐1a); 138.40 (C‐2); 137.13 (C‐14); 123.51 (C‐4a); 122.94 (C‐6); 120.78 (C‐5a); 131.44 (C‐16, C‐18); 126.54 (C‐15, C‐19); 125.28 (C‐7); 120.80 (C‐4); 119.80 (C‐17); 119.68 (C‐5); 118.59 (C‐3); 112.40 (C‐8); 110.20 (C‐1); 45.44 (C‐10); 18.32 (C‐11).

**FT‐IR** (ATR in solid, ν cm^−1^): 3370m; 3228m; 3185m; 3031w; 2972w; 1681m; 1593m; 1525vs; 1480sh; 1339m; 1256m; 1158s; 1066s; 1015m; 867w; 803m; 712m.

Chemical formula C_22_H_17_BrClN_3_OS, exact mass 484.99642, **HRMS** (APCI+, MeCN), m/z (%): 488.00178 (76) [M+H]^+^, 454.01434 (5) [M‐S‐H]^+^, 429.01916 (7), 314.02801 (14) [C_16_H_11_ClN_2_OS]^+^, 273.07936 (100) [C_15_H_14_ClN_2_O]^+^, 255.04506 (6) [C_15_H_10_ClNO]^+^, 228.05789 (67) [C_14_H_11_ClN]^+^, 214.08999 (10), 123.07919 (15), 82.05234 (6).

#### 3.3.6 2‐(6‐Chloro‐9*H*‐carbazol‐2‐yl)‐*N*‐((4‐iodophenyl)carbamothioyl)propanamide (1d) (m.p. 189.6–198.4 °C; Yield 76%)

^**1**^ **H‐NMR** (500 MHz, dmso‐d6, *δ* ppm, *J* Hz): 12.39 (CS–HN–Ar); 11.70 (s, H‐9); 11.43 (CO–NH–CS); 8.18 (d, H‐5, 1.9); 8.13 (d, H‐4, 8.2); 7.72 (d, 2H, H‐16, H‐18, 8.5); 7.54 (bs, 1H, H‐1); 7.49 (d, 1H, H‐8, 8.6); 7.44 (d, 2H, H‐15, H‐19, 8.5); 7.37 (dd, 1H, H‐7, 2.1, 8.6); 7.22 (bd, 1H, H‐3, 8.2); 4.24 (q, H‐10, 7.0); 1.50 (d, H‐11, 7.0).

^**13**^ **C‐NMR** (125 MHz, dmso‐d6, *δ* ppm): 178.79 (C‐13); 176.05 (C‐12); 140.41 (C‐8a); 138.57 (C‐1a); 138.33 (C‐2); 137.49 (C‐14); 91.27 (C‐17); 137.41 (C‐16, C‐18); 126.52 (C‐15, C‐19); 125.39 (C‐7); 123.54 (C‐4a); 123.04 (C‐6); 120.95 (C‐4); 120.84 (C‐5a); 119.81 (C‐5); 118.70 (C‐3); 112.46 (C‐8); 110.20 (C‐1); 45.50 (C‐10); 18.38 (C‐11).

**FT‐IR** (ATR in solid, ν cm^−1^): 3370m; 3230w; 3033w; 2966w; 1680m; 1589m; 1529vs; 1476sh; 1340m; 1259m; 1161m; 1066s; 1017s; 868w; 804vs; 714m.

Chemical formula C_22_H_17_ClIN_3_OS, exact mass 532.98255, **HRMS** (APCI+, MeCN), m/z (%): 533.99115 (100) [M+H]^+^, 500.00371 (10) [M‐S‐H]^+^, 475.00860 (65), 359.00888 (40), 315.03661(11) [C_16_H_12_ClN_2_OS]^+^, 273.08011 (60) [C_15_H_14_ClN_2_O]^+^, 228.05855 (59) [C_14_H_11_ClN]^+^, 219.96294 (10) [C_6_H_7_IN]^+^.

### 3.4 Antimicrobial Activity

The strains used for testing were *S. aureus* ATCC 25923, *E. faecalis* ATCC 19433, *P. aeruginosa* ATCC 27853, *K. pneumoniae* ATCC 13368, and *C. albicans* ATCC 10231.

#### 3.4.1 Quantitative Evaluation of Antimicrobial Activity

Quantitative analysis was performed using the serial binary microdilution method in liquid medium (Tryptone Soy Broth for bacteria, Sabouraud for yeasts) in 96‐well plates. Stock solutions of 10 mg/mL were prepared in DMSO, and the concentration range for each sample was 5000–0.031 µg/mL. Simultaneously, a solvent control, DMSO, was performed under the same working conditions. Each well was inoculated with 10 μL of microbial suspension, adjusted to a density of 1.5 × 108 CFU/mL for bacteria and 1.5 × 106 CFU/mL for yeasts, prepared from 18 to 24 h cultures. After 24 h at 37 °C, microbial growth and multiplication were evaluated both macroscopically and by monitoring optical density at 620 nm to determine the MIC values (the last concentration at which no microbial growth was observed, or at which the absorbance value decreased to the level of the sterility control). The obtained data were analyzed using the Inhibitor vs Response ‐ Variable Slope (Four Parameters) analysis function with the help of Prism GraphPad 10.0 software, in order to calculate the IC50 (the concentration of the sample that inhibits microbial growth by 50% compared to untreated cultures).

#### 3.4.2 Evaluation of Microbicidal Activity

To determine the MMCs, 5 µL of culture was taken from each well and spread onto solid medium. The plates were incubated for 20–24 h at 37 °C for bacteria and yeasts. The last concentration at which no microbial growth was observed was considered the MMC.

#### 3.4.3 Microbial Adherence

Following the quantitative analysis of antimicrobial activity, microbial adherence was evaluated after methanol fixation and staining with crystal violet (0.1%). The absorbance of the biomass stained with crystal violet and resuspended in 33% acetic acid was measured at 490 nm. Bacterial adhesion to the inert surface was evaluated by inverted optical microscopy (Nikon Eclipse TS2R) using a 10× objective.

#### 3.4.4 Statistical Analysis

Data are expressed as means ± standard deviation (SD) (determined by duplicate analysis). Statistical analysis was performed using GraphPad Prism 10.0. For comparing the IC50 values of the newly synthesized compounds and the solvent, one‐way ANOVA was applied, followed by Tukey's multiple comparison test with a single combined variance. The significance level was set at *p* < 0.05.

### 3.5 Antioxidant Activity

#### 3.5.1 DPPH Assay

The DPPH test was performed using the method reported by Corbu et al. [59], with some adjustments. The reaction mixture consisted of 500 µL of sample/standard in DMSO and 500 µL of 0.3 mM DPPH radical solution. Absorbance was measured at *λ* = 517 nm after 25 min of incubation in the dark and centrifugation for 5 min at 10,000 rpm to separate the precipitate. The results were expressed as Trolox equivalents in µM per mg of compound. All experiments were performed in triplicate. The DPPH inhibition percentage was calculated using the following formula:

$$\text{DPPH}\;\mathrm{inhibition}\;\left(\%\right)=\frac{A‐B}{A}\times 100$$

where *A* represents the absorbance of the solvent control and *B* represents the absorbance of the samples. The concentration required to achieve 50% inhibition of the DPPH radical (IC50) was determined using linear regression analysis of concentration versus the percentage of DPPH inhibition.

#### 3.5.2 *Ferric Reducing Antioxidant Power* (*FRAP*) *Assay*

The FRAP assay was performed according to the protocol described by Corbu et al. [59] with a few adjustments. The reaction mixture consisted of 475 µL of FRAP reagent and 25 µL of sample/standard. Absorbance was measured at *λ* = 593 nm after 25 min of incubation at 37 °C in the dark and 5 min of centrifugation at 10,000 rpm. The Trolox calibration curve included values ranging from 25 to 250 µM (*R* ^2^ = 0.9969). The results were expressed as Trolox equivalents in µM per mg of compound.

#### 3.5.3 *Cupric Reducing Antioxidant Capacity* (*CUPRAC*) *Assay*

The CUPRAC test was performed using the method described by Bostiog et al. [60]. A volume of 120 μL of sample/standard solutions with different concentrations was mixed with 100 μL of CuCl_2_ solution (10 mM) and 100 μL of neocuproine (7.5 mM). After 25 min of incubation and centrifugation at 10,000 rpm for 5 min, the absorbance was measured at 450 nm. The calibration curve was obtained using Trolox at concentrations ranging from 0.100 to 1.5 mM (*R* ^2^ = 0.9999). The results were expressed as Trolox equivalents in µM per mg of compound.

#### 3.5.4 *Trolox Equivalent Antioxidant Capacity* (*TEAC*) *Assay*

The Trolox Equivalent Antioxidant Capacity (TEAC) assay was performed according to the method described by Corbu et al. [53], with minor modifications. An ABTS+ stock solution was prepared by mixing a 7 mM ABTS solution with 2.45 mM potassium persulfate. Before use, the mixture was kept in the dark for 12–16 h to allow for the complete formation and stabilization of the ABTS^+^ cationic radical. Diluting ABTS+ with methanol resulted in an usable solution with an absorbance of approximately 0.70. The reaction mixture included 100 μL of sample/standard and 900 μL of working ABTS+ solution. It was incubated for 25 min in the dark and centrifuged for 5 min at 10,000 rpm. The standard curve was linear in the concentration range of 20–150 µM Trolox (*R* ^2^ = 0.9974). The results were expressed as µM Trolox per mg of substance.

### 3.6 In Vitro Hemocompatibility Evaluation

The hemolytic index was evaluated using a spectrophotometric method [61]. A volume of 9 mL of sheep blood was collected in acid citrate dextrose (ACD) tubes and centrifuged for 10 min at 5000 rpm and 4 °C. The supernatant was removed, and the sediment was washed three times with phosphate‐buffered saline (PBS) solution (0.2 M, pH 7.4) before being resuspended in sterile saline solution (0.9%). Additionally, 100 µL of the stock solutions from each sample (1 mg/mL in DMSO) was added to 400 µL of a 10% erythrocyte suspension. The mixtures were incubated for 1 h at 37 °C. The samples were centrifuged at 5000 rpm for 10 min, and the absorbance of the supernatant was measured at 540 nm. To achieve 100% (*A* ^+^) and 0% (*A* ^−^) hemolysis controls, an erythrocyte suspension was incubated with 1% (v/v) Triton X‐100 and 0.9% saline solution, respectively. A solvent control was also used. Each compound was tested in triplicate, and the inhibitory activity was expressed as a percentage of hemolysis. Hemolysis (%) was calculated using the equation below:

$$\text{Hemolysis }\left(\%\right)=\frac{A_{\text{sample}}-A^{-}}{A^{+}-A^{-}}\cdot 100$$

#### 3.6.1 Antihemolytic Activity by AAPH‐Induced Oxidative Hemolysis

A previously published spectrophotometric method was used to assess the newly synthesized compounds’ antihemolytic capability in accordance with Geana et al. [62]. Nine milliliters of ram blood was collected in ACD tubes and centrifuged at 5000 rpm for 10 min. After the supernatant was discarded, the pellet was resuspended in saline solution (0.9%) and rinsed three times with PBS (0.2 M, pH 7.4). In addition, 50 µL of each chemical (5, 2.5, and 1.25 mg/mL in PBS, pH = 7.4) was added to 200 µL of 10% erythrocyte suspension. After 20 min of incubation at 37 °C, 100 µL of AAPH (2,2′‐azobis(2‐amidinopropane) dihydrochloride) (200 mM final concentration) was added to the reaction mixture to promote membrane lipid oxidation. The mixes were incubated for 4 h at 37 °C. After centrifuging the samples for 10 min at 5000 rpm, the absorbance of the supernatant was measured at 540 nm. The hemolysis in the AAPH‐treated negative control (*A*_AAPH_) was set at 100%, and this has been used to calculate the relative hemolysis. Instead of AAPH, the positive control was PBS (*A*_PBS_). Each set of experiments was performed in triplicate, and inhibitory activity was calculated, expressed herein as percent inhibition of hemolysis. A stock solution of ascorbic acid was treated in a similar manner to the method employed for the samples. The hemolysis (%) was calculated as follows:

$$\text{Antihemolytic effect}\;(\%)=100-\frac{A_{\text{sample}}-A_{\mathrm{PBS}}}{A_{\text{AAPH}}-A_{\mathrm{PBS}}}\cdot 100$$

### 3.7 Cytotoxicity Evaluation

Cell viability was assessed using the CellTiter Aqueous One Solution Cell Proliferation Assay (MTS) (Promega, Madison, WI, United States), according to the manufacturer's instructions. The following cell lines were used: MG‐63 human osteosarcoma cells derived from bone tissue (CRL‐1427), A‐431 human epidermoid carcinoma cells isolated from skin (CRL‐1555), HT‐29 colorectal adenocarcinoma cells (HTB‐38), and HCT‐8 human ileocecal adenocarcinoma cells (CCL‐244). For the assay, binary serial dilutions of the compounds were prepared in the range 500–3.9 µg/mL in 96‐well plates; cells were then seeded at 7500 cells/well and maintained at 37 °C in a humidified atmosphere with 5% CO_2_. After 72 h of incubation, 20 µL of MTS reagent was added to each well, and the plates were incubated for 2 h at 37 °C in a humidified atmosphere with 5% CO_2_. Absorbance was measured at 490 nm using a microplate reader. Cell viability was expressed as percentage relative to untreated controls, and IC50 values were calculated using the Quest Graph IC50 Calculator [63].

### 3.8 Bioinformatics

#### 3.8.1 Molecular Docking Study

Molecular docking was performed using protein structures obtained from the Protein Data Bank (PDB) under accession codes 3TTZ [57], crystal structure of a topoisomerase (*S. aureus*), and 4EEQ, crystal structure DNA ligase (*E. faecalis*). Both structures were downloaded in PDB format and processed locally using UCSF ChimeraX to remove heteroatoms, water molecules, and existing ligands. The proteins were further prepared for docking using the DockPrep tool in ChimeraX [64], which involved removing nonstandard residues and optimizing protonation states (adding hydrogen atoms) [65].

The 2D structures of the synthesized compounds were drawn, and their SMILES strings were converted into 3D .sdf files using OpenBabel [66]. Subsequently, molecular geometry optimization was performed using Avogadro and the optimized structures were saved as .sdf files.

Molecular docking simulations were carried out using AutoDock Vina within the PyRx interface [67]. Prior to docking, the compounds underwent energy minimization and were converted into the .pdbqt format required by the software.

Docking analyses were performed separately for each protein for an exhaustiveness of 50. For the 3TTZ protein, a grid box with dimensions of *X*: 66.68, *Y*: 71.43, and *Z*: 67.55 Å was defined to ensure complete coverage of the protein. For the 4EEQ structure, the same molecular docking protocol was applied, utilizing a similarly defined grid box with dimensions of *X*: 50.4444, *Y*: 49.5069, and *Z*: 75.9890 Å.

To visualize the docking results, we used Discovery Studio Visualizer (Discovery Studio 24.1.0.23298; Dassault Systèmes BIOVIA, Discovery Studio Visualizer, version 17.2l; Dassault Systèmes: San Diego, CA, United States, 2020) to highlight the interactions at the amino acid residue level between the synthesized compounds and the two target proteins, topoisomerase (3TTZ) and DNA ligase (4EEQ). This analysis is crucial for elucidating the binding modes and identifying the key residues involved in ligand recognition.

#### 3.8.2 Molecular Modeling and 3D Structure Generation

The thiourea derivatives were modeled using Spartan software [68]. Initially, 2D molecular structures were constructed, followed by introduction of specific substituents and completion of all valences with hydrogen atoms. The optimized 2D structures were then converted into three‐dimensional space to generate accurate 3D molecular geometries suitable for subsequent computational analyses, including pharmacokinetic prediction. SMILES files were generated for each compound and used in subsequent bioinformatics analyses. Molecular minimization was performed using the Hamiltonian method with the MMFF94x force field at a gradient of 0.05. After identification of the minimum‐energy conformations, Gasteiger partial charges (PEOE) were applied to each molecule.

To assess the drug‐like properties of the newly synthesized compounds, compliance with Lipinski's rule of five and Veber's rules was evaluated using SwissADME [69]. PAINS (pan‐assay interference compounds), Brenk structural alerts, lead‐likeness, and synthetic accessibility scores were also evaluated using the same platform.

Pharmacokinetic properties were predicted using Deep‐PK [60]. The estimated parameters included the following: (i) Caco‐2 absorption, human intestinal absorption, P‐glycoprotein inhibitor/substrate status, and skin permeability; (ii) BBB/CNS penetration and plasma protein binding; and (iii) OATP1b1, OATP1b3, clearance, OCT2, and half‐life.

The pharmacogenomic profiles of compounds as substrates or inhibitors of the major cytochromes CYP1a2, CYP2C19, CYP2C9, CYP2D6, and CYP3A4 were predicted using Deep‐PK [70].

Toxicity profiles were assessed using ProTox 3.0, which provides predictions for acute toxicity, organ toxicity, toxicological endpoints, molecular‐initiating events, and Tox21 pathways [71].

The prediction of compound toxicities is an important component of drug design and development. Computational toxicity estimations are faster than experimental determination of toxic doses in animals and can help reduce animal testing. Using the advanced machine‐learning methods included in ProTox 3.0 [62], we evaluated a broad panel of toxicity endpoints. ProTox 3.0 incorporates molecular similarity, fragment propensities, most frequent features, and machine‐learning methods based on cluster cross‐validation, with 61 models for prediction of toxicity endpoints such as acute toxicity, organ toxicity, toxicological endpoints, molecular‐initiating events, metabolism, adverse‐outcome (Tox21) pathways, and toxicity targets.

## 4 Conclusion

Continuing our efforts to develop new agents against resistant microbial pathogens, we synthesized a series of carbazole‐based *N*‐(arylcarbamothioyl)propanamides incorporating carbazole, propionic acid, and thiourea pharmacophores. The compounds exhibited selective antimicrobial activity, particularly against *S. aureus* and *C. albicans*, together with antioxidant and antihemolytic properties. Among them, compound **1a** emerged as the most promising candidate, combining antimicrobial efficacy with strong antioxidant activity, erythrocyte membrane protection, and a favorable safety profile. In contrast, compound **1b** showed the highest cytotoxic activity, especially against human epidermoid carcinoma cells, making it a suitable candidate for further mechanistic studies. Overall, these findings identify *N*‐(arylcarbamothioyl)propanamides as a promising class of bioactive molecules and provide a basis for future structural optimization and mechanistic investigations.

## Conflicts of Interest

The authors declare no conflicts of interest.

## Supporting information

Supplementary Material

## Data Availability

The data that support the findings of this study are available from the corresponding author upon reasonable request.
