# Supplementary material for: Novel Carbazole–Acylthiourea Derivatives: Synthesis, Antimicrobial, Antioxidant, Hemocompatibility, Cytotoxicity, and ADME‐Tox Profiling
Source: ChemistryOpen. 2026 Oct 12;15(11):e70258. doi: 10.1002/open.70258 (PMC13657261; doi:10.1002/open.70258)
Supplement: Supplementary file 1 [file OPEN-15-e70258-s001.pdf]

Supporting Information

Novel Carbazole-Acylthiourea Derivatives: Synthesis, Antimicrobial, Antioxidant, Hemocompatibility, Citotoxicity and ADME-Tox Profiling

Carmen Limban[1], Ioana Cristina Marinaș[2],* Mariana Carmen Chifiriuc[2,3,4], Miron Teodor Căproiu[5], Florea Dumitrașcu[5], Erzsébet-Eleonóra Kapronczai[6], Irina Zarafu[2,7], Diana Camelia Nuţă[1], Petre Ioniță[7], Speranța Avram[8], Cătălina Mareș[8], Andra Maria Păun[8], Bogdan Mihai Cristea[9], Carmellina Daniela Bădiceanu[1], Ilinca Margareta Vlad[1], Maria Coandă[1], Delia Laura Popescu[10], Coralia Bleotu[2,11]

[1]Department of Pharmaceutical Chemistry, Faculty of Pharmacy, "Carol Davila" University of Medicine and Pharmacy, 6 Traian Vuia, 020956, Bucharest, Romania
[2]Research Institute of the University of Bucharest (ICUB), University of Bucharest, 90 Panduri Street, 050663, Bucharest, Romania
[3]Department of Microbiology, Faculty of Biology, University of Bucharest, 060101 Bucharest, Romania
[4]Romanian Academy, 010071 Bucharest, Romania
[5]"C.D. Nenitzescu" Institute of Organic and Supramolecular Chemistry of the Romanian Academy, 202B Splaiul Independenţei, 060023, Bucharest, Romania
[6]Supramolecular Organic and Organometallic Chemistry Centre, Department of Chemistry, Faculty of Chemistry and Chemical Engineering, Babeș-Bolyai University, 11 Arany János, 400028, Cluj-Napoca, Romania
[7]Department of Organic Chemistry, Biochemistry and Catalysis, Faculty of Chemistry, University of Bucharest, 4-12 Regina Elisabeta, 030018, Bucharest, Romania
[8]Department of Anatomy, Animal Physiology, and Biophysics, Faculty of Biology, University of Bucharest, 36-46 M. Kogălniceanu Boulevard, 050107, Bucharest, Romania
[9] Preclinical Department 2, Morphological Sciences, Faculty of Medicine, Carol Davila University of Medicine and Pharmacy Bucharest, 020021 Bucharest, Romania; bogdan.cristea@umfcd.ro
[10]Faculty of Interdisciplinary Studies, University of Bucharest, 36-46 M. Kogălniceanu Boulevard, 050107, Bucharest, Romania, delia.popescu@chimie.unibuc.ro
[11]Department of Cellular and Molecular Pathology, Ștefan S. Nicolau Institute of Virology, 285 Mihai Bravu Ave., 030304 Bucharest, Romania

Correspondence: Ioana Cristina Marinaș (ioana-cristina.marinas@icub.unibuc.ro)

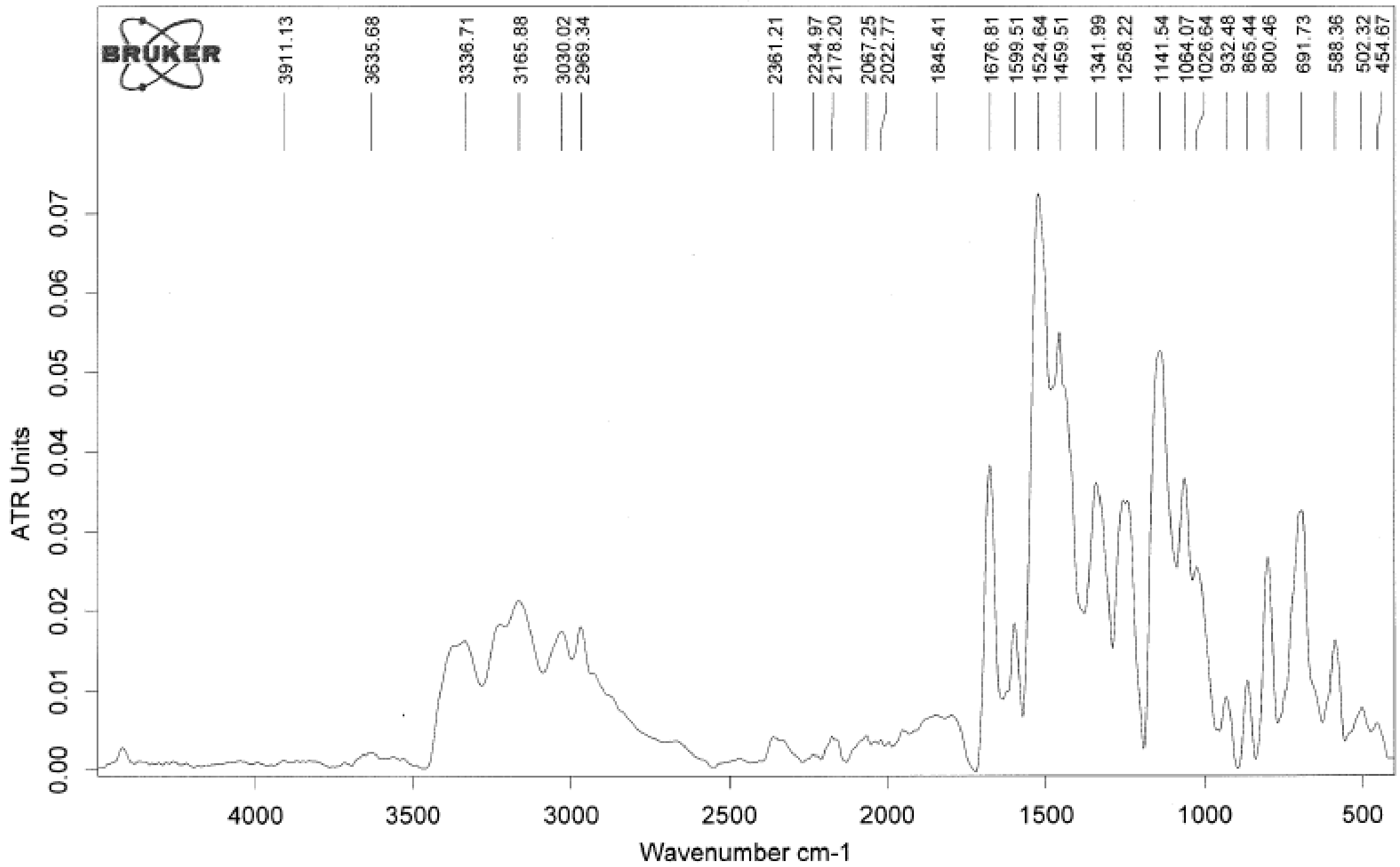

Figure S1. The IR spectrum of compound **1a**

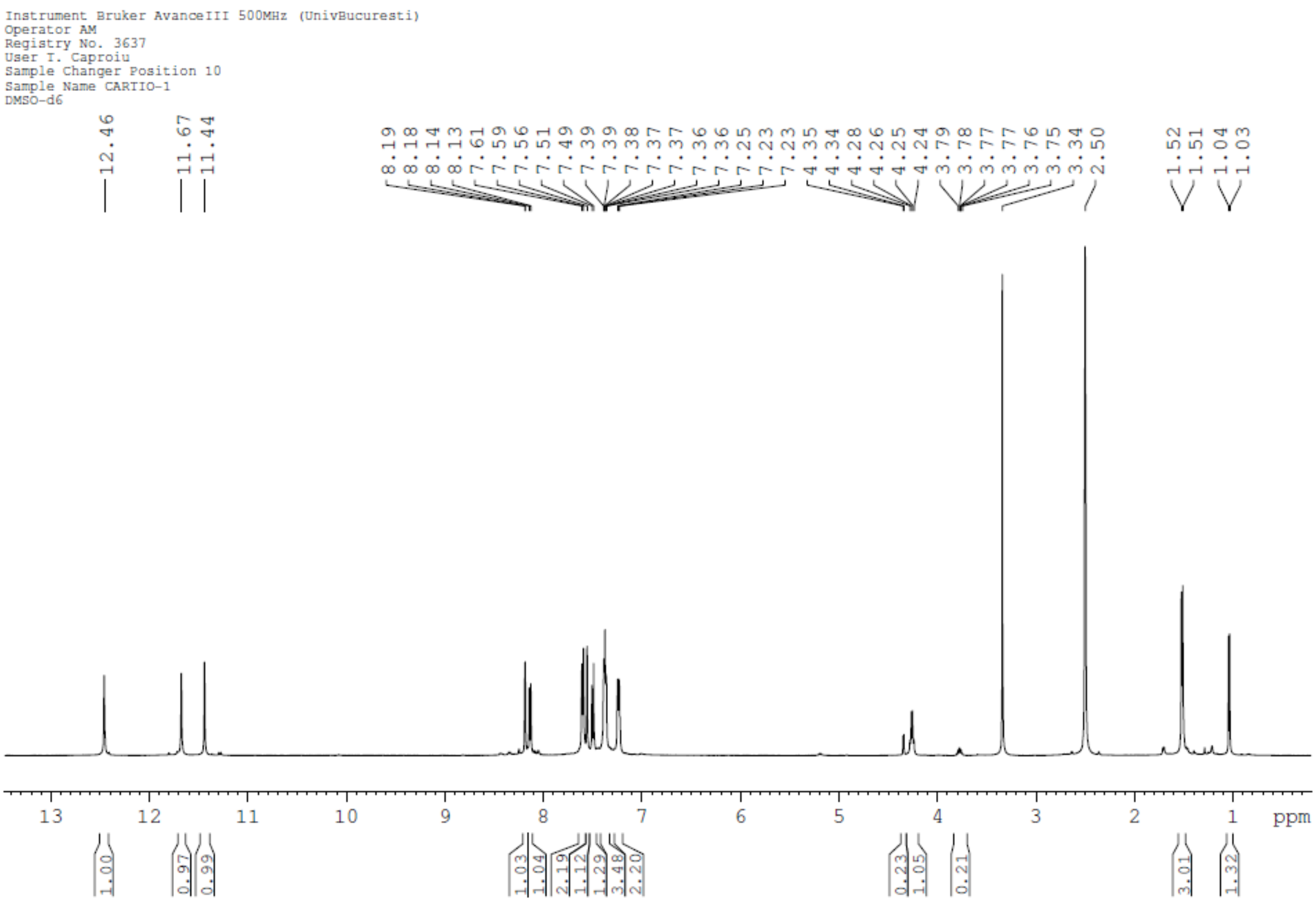

Figure S2. The ^{1}H-NMR spectrum of compound **1a**

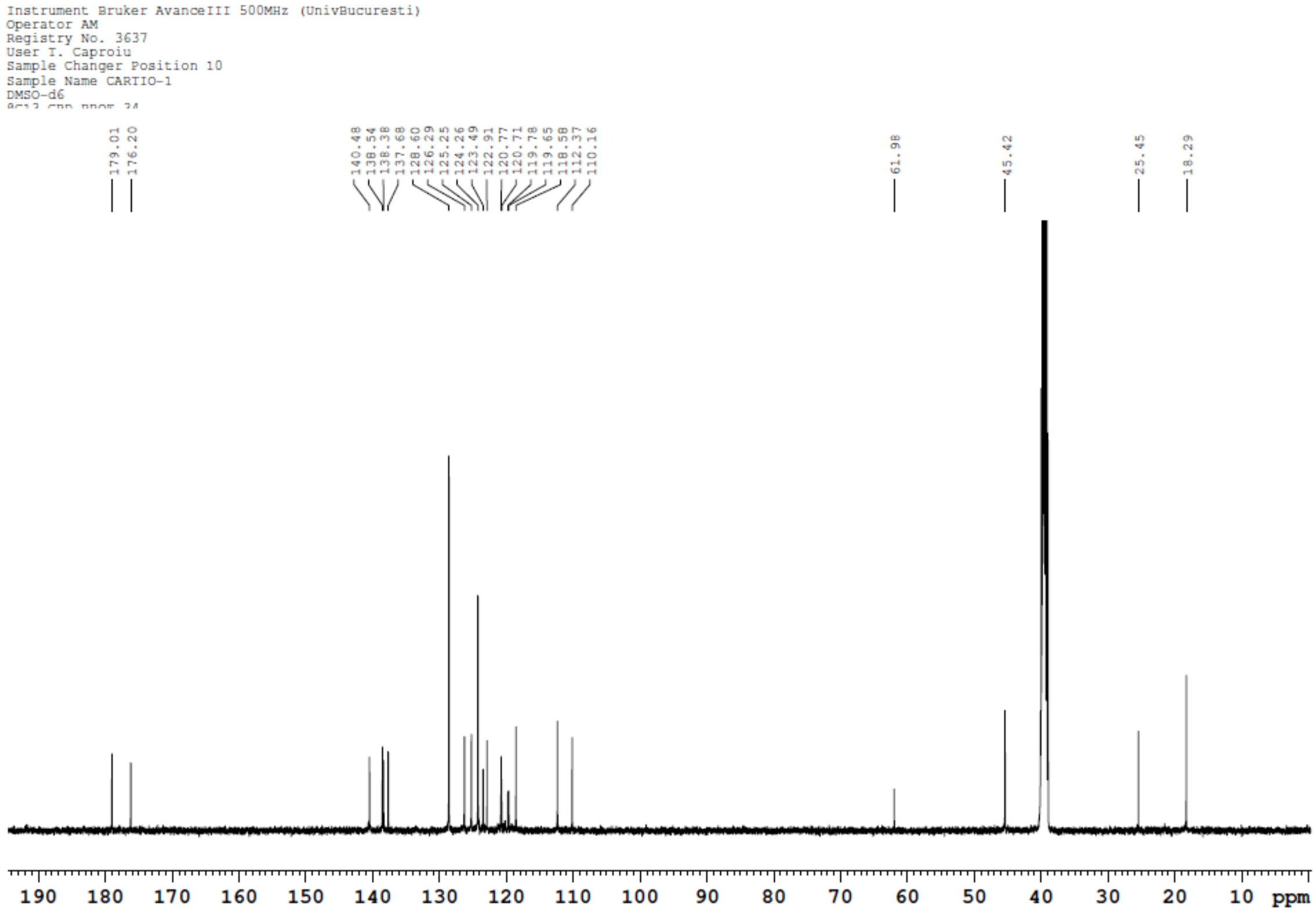

Figure S3. The ^{13}C-NMR spectrum of compound **1a**

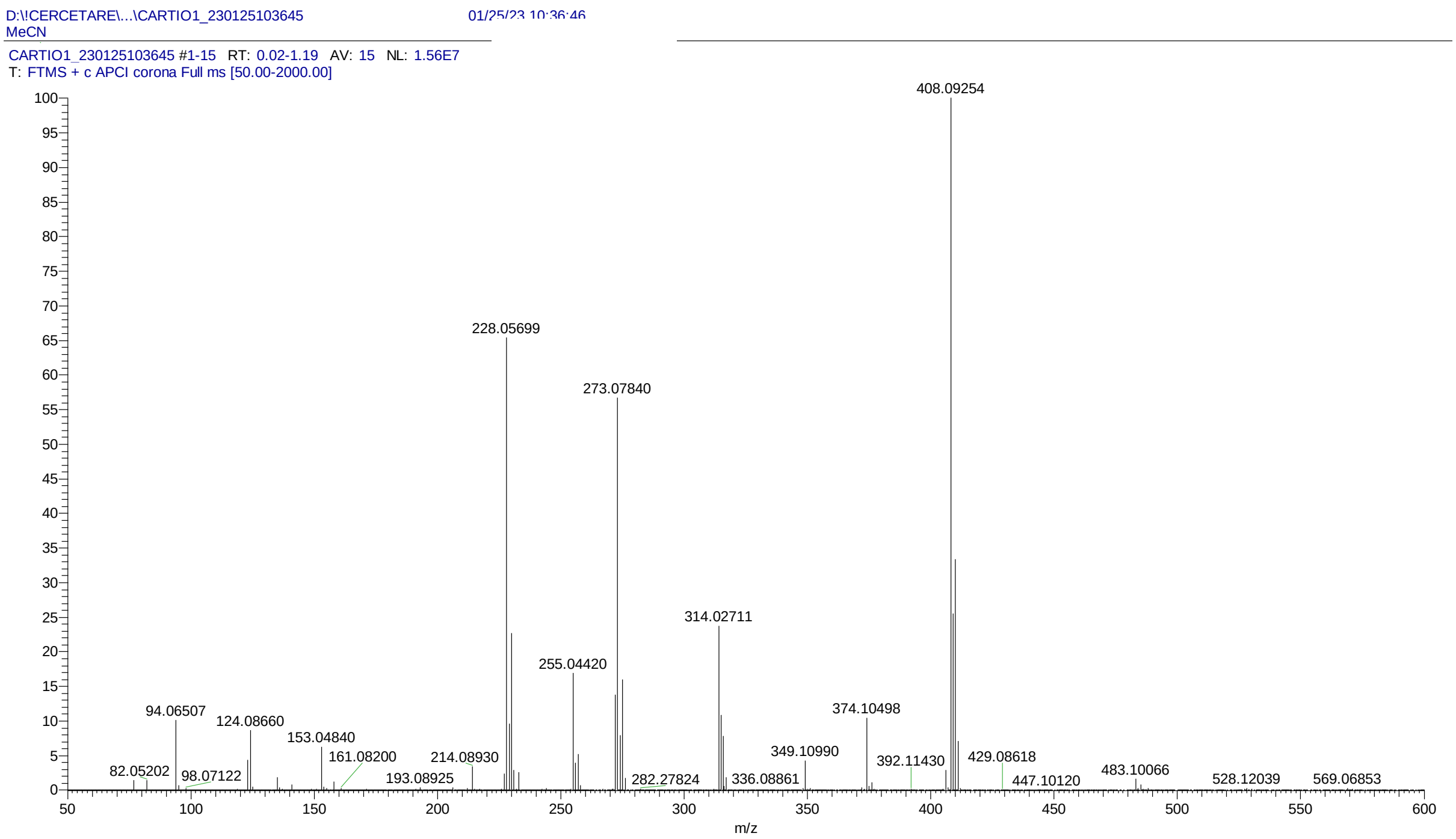

Figure S4. The APCI+ MS spectrum of **1a** in MeCN

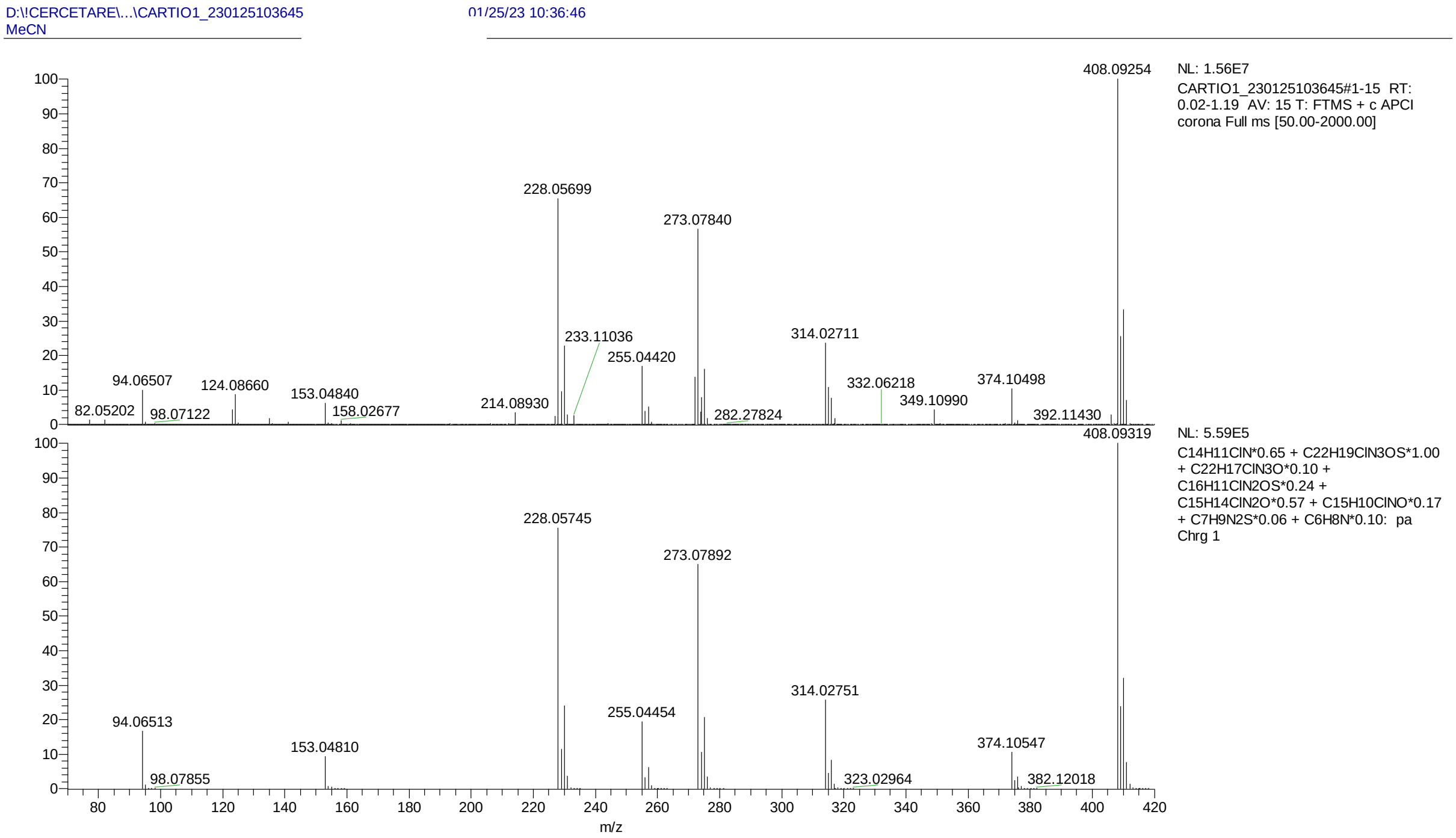

Figure S5. The experimental (up) and calculated (down) APCI+ MS spectra of **1a**

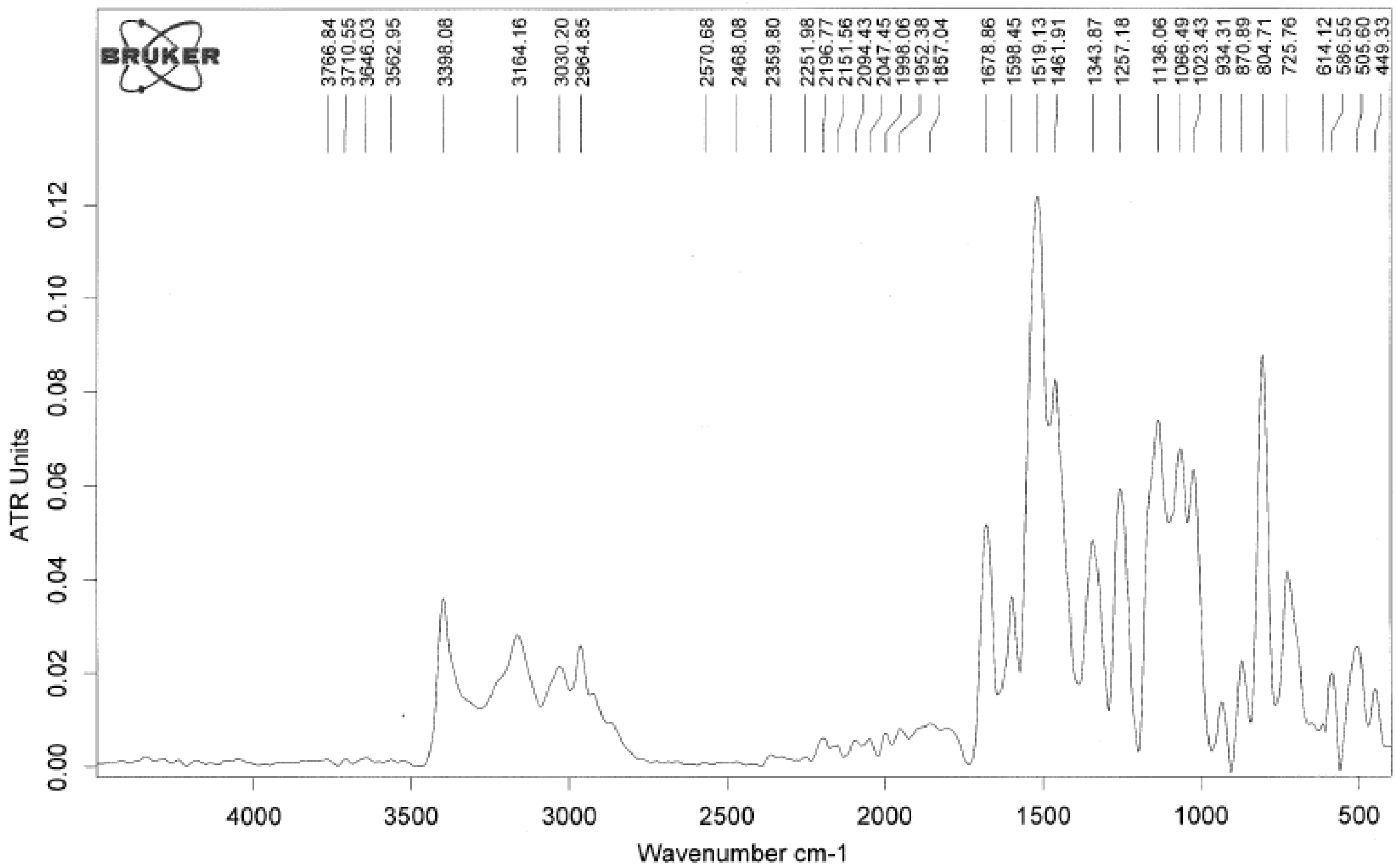

Figure S6. The IR spectrum of compound **1b**

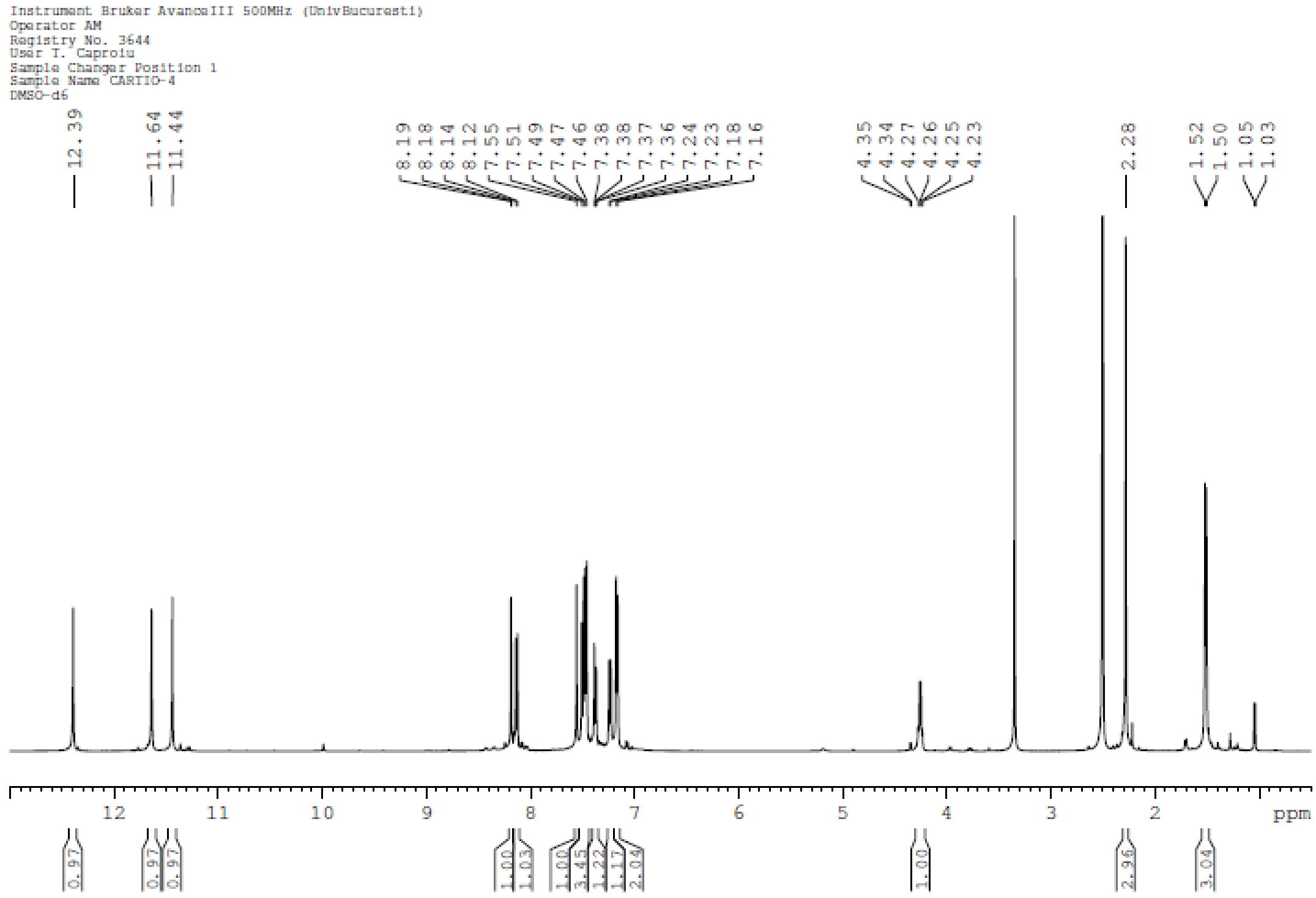

Figure S7. The ^{1}H-NMR spectrum of compound **1b**

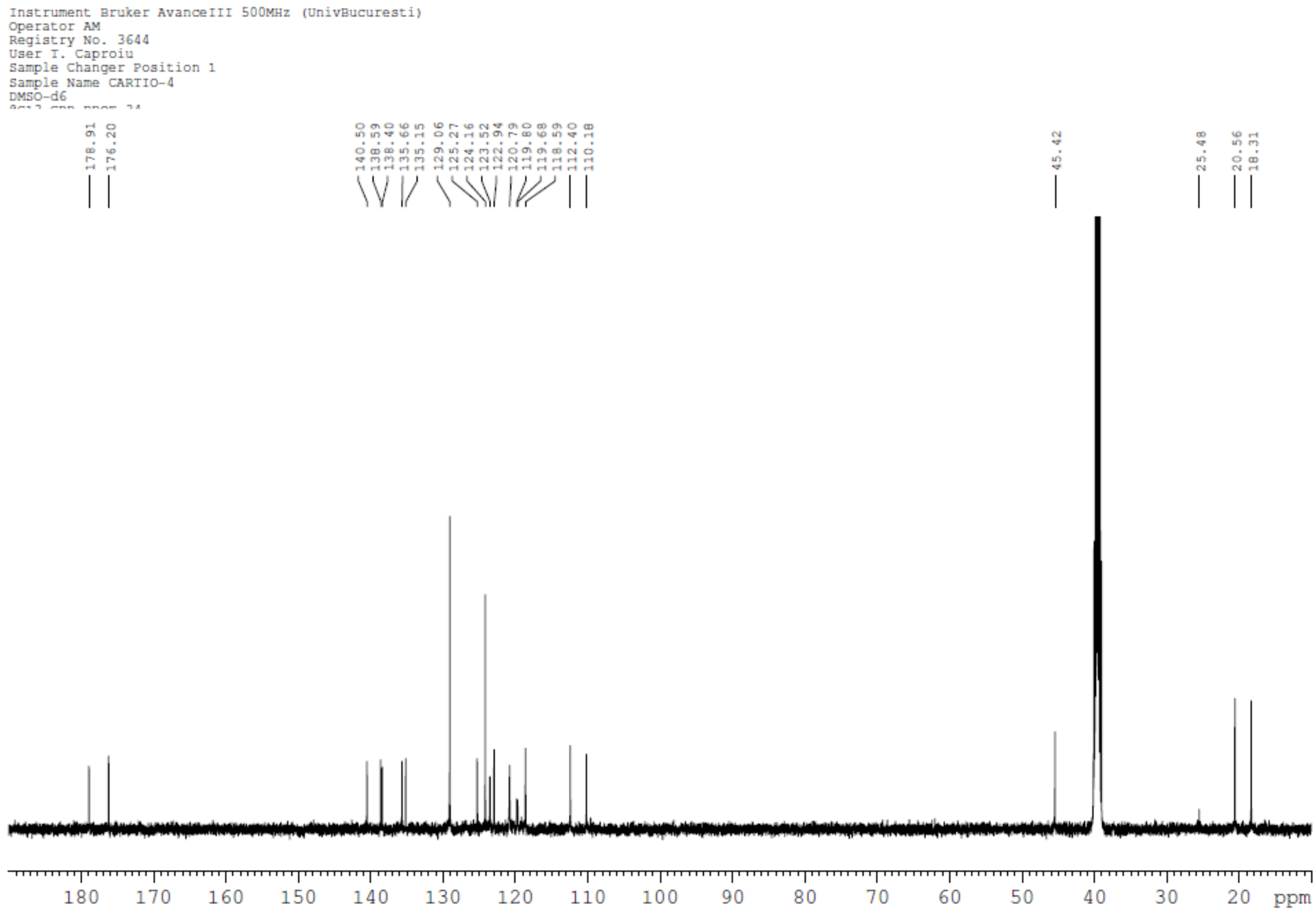

Figure S8. The ^{13}C-NMR spectrum of compound **1b**

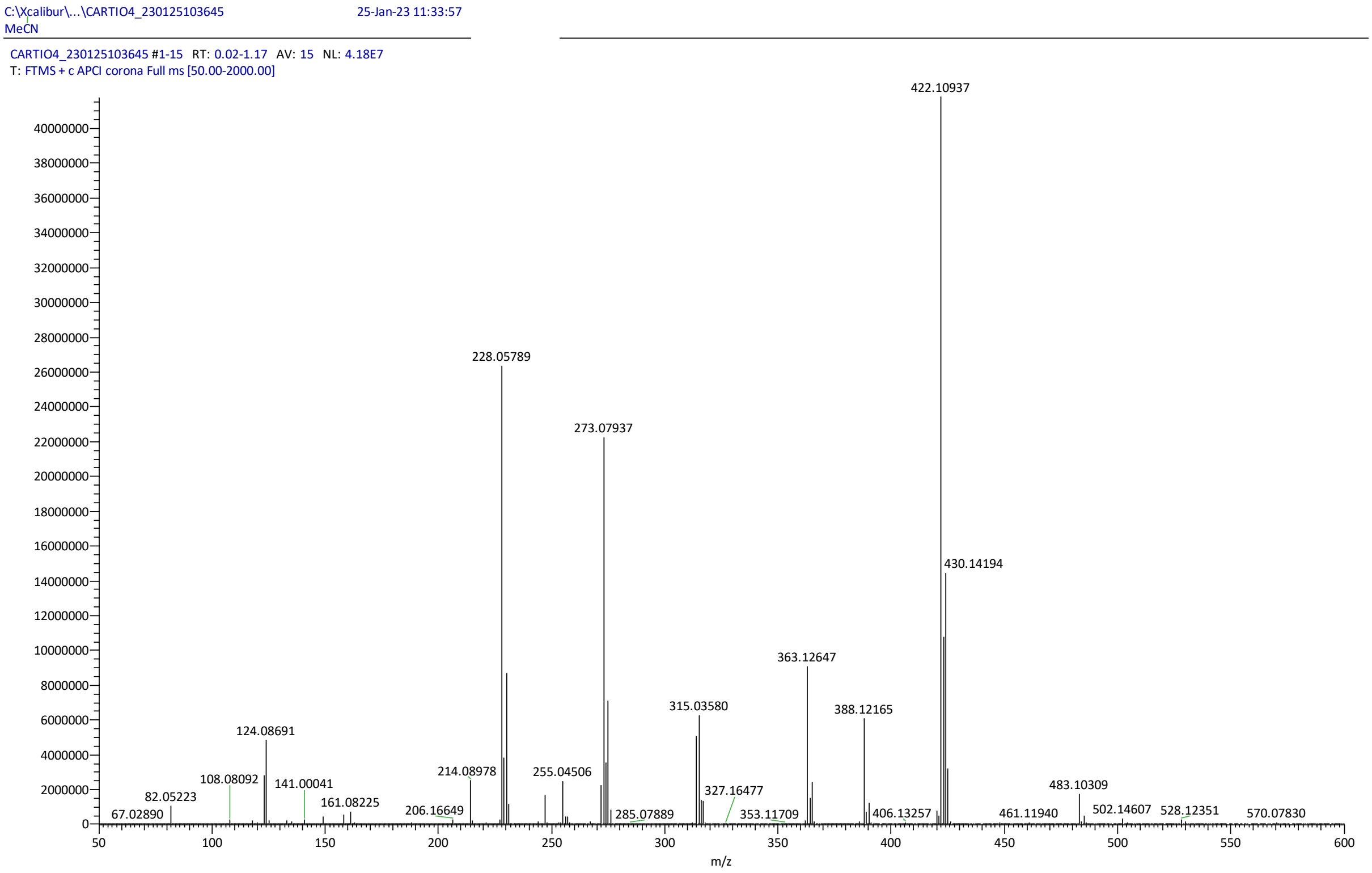

Figure S9. The APCI+ MS spectrum of **1b** in MeCN

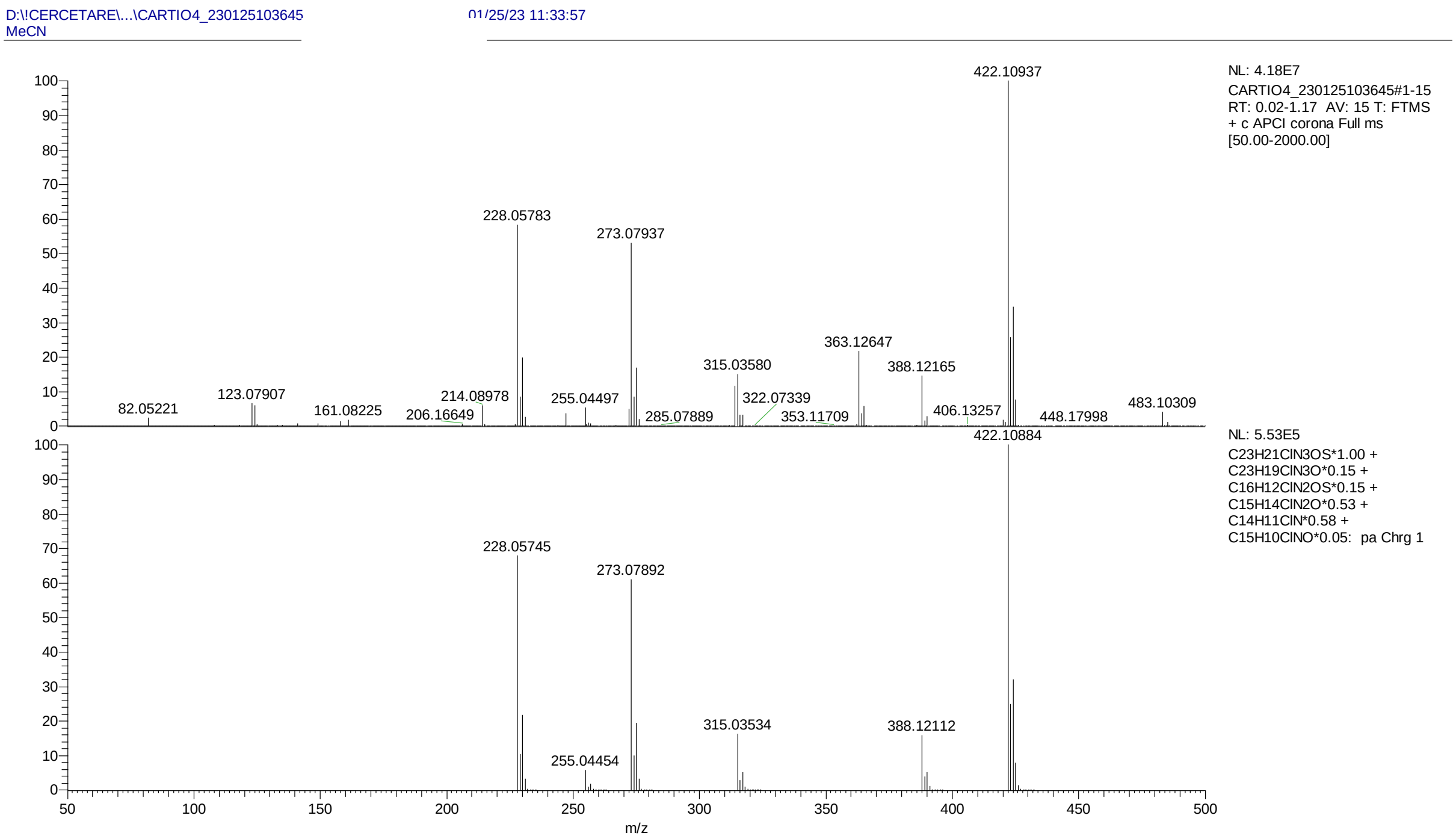

Figure S10. The experimental (up) and calculated (down) APCI+ MS spectra of **1b**

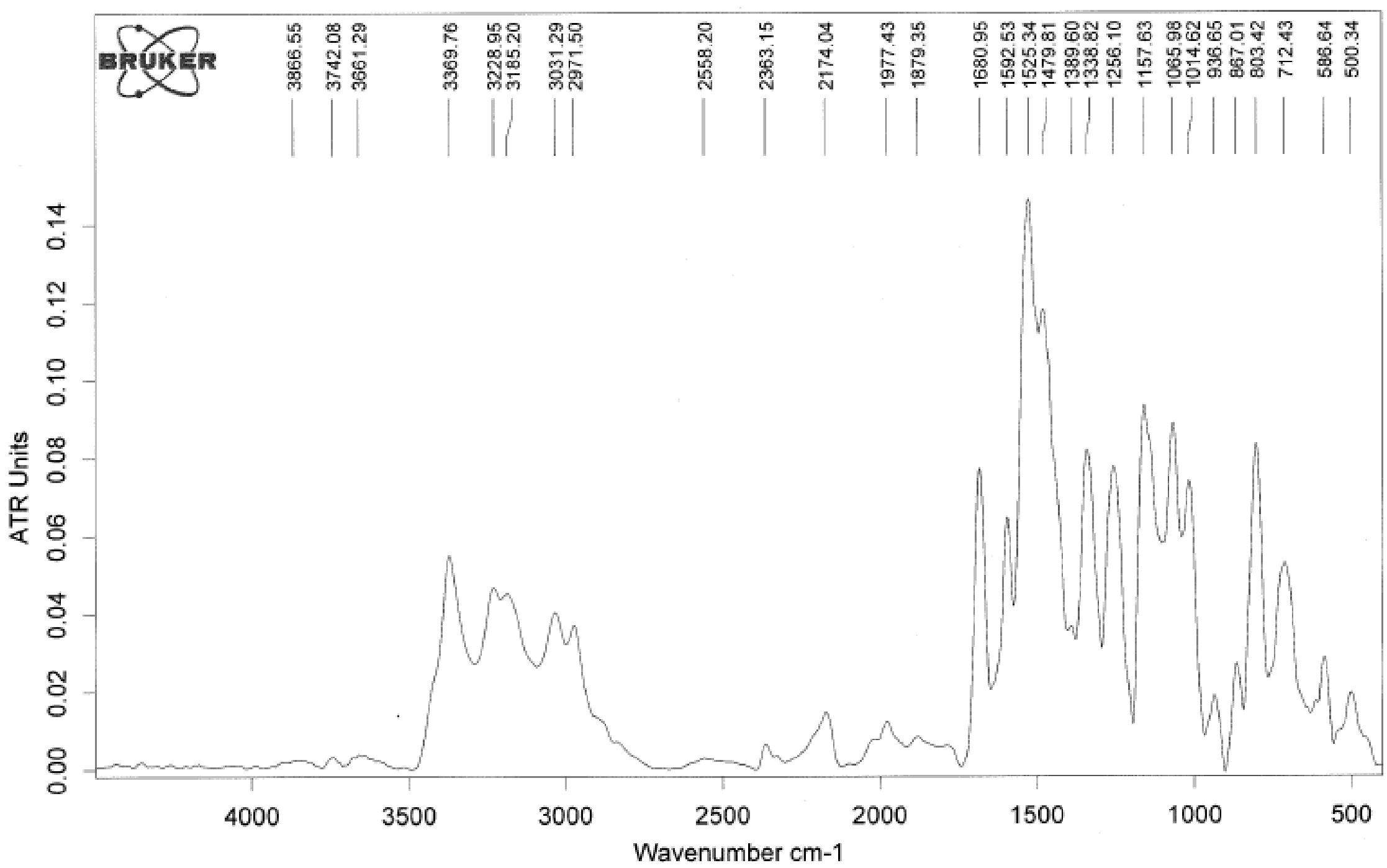

Figure S11. The IR spectrum of compound **1c**

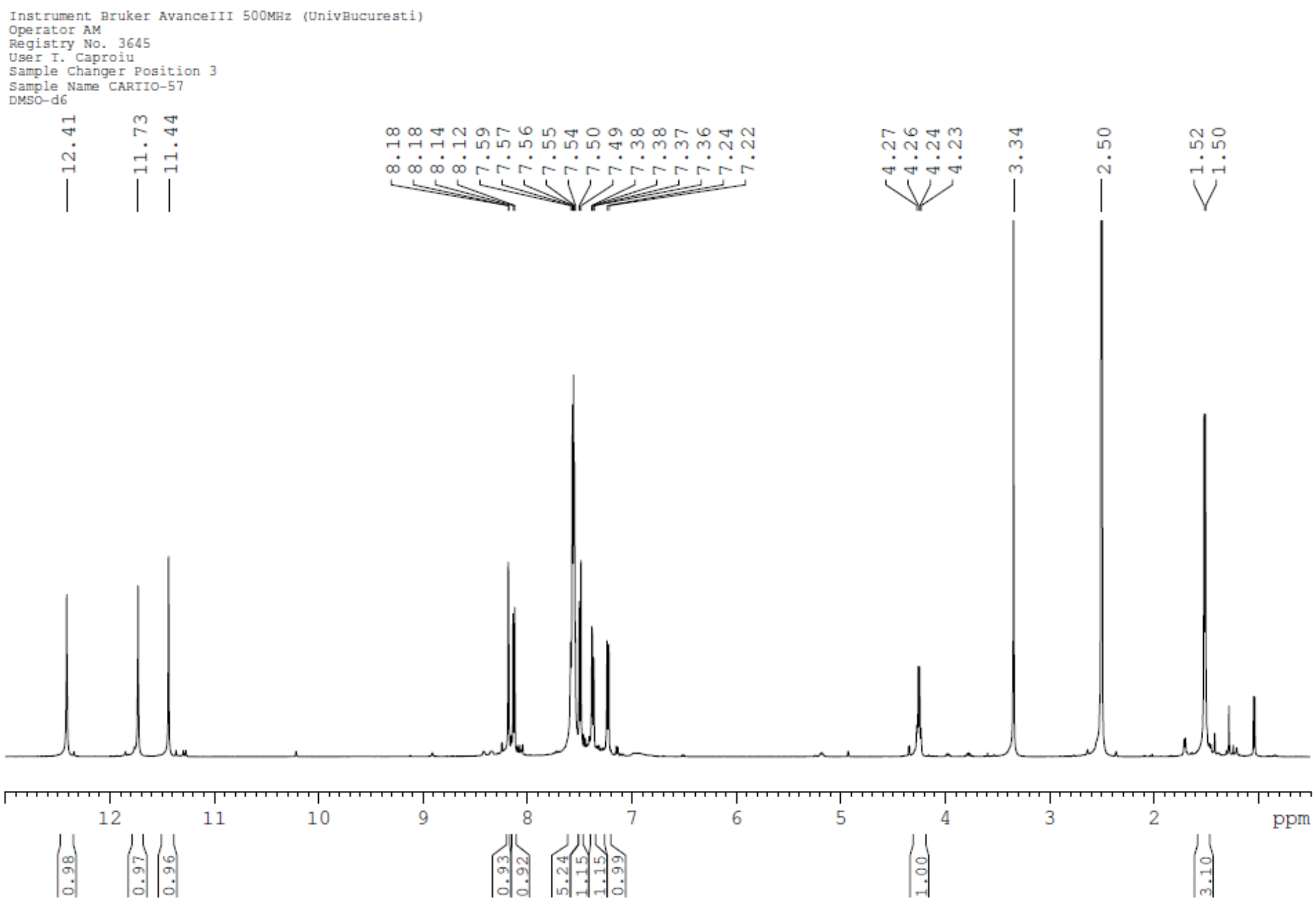

Figure S12. The ^{1}H-NMR spectrum of compound **1c**

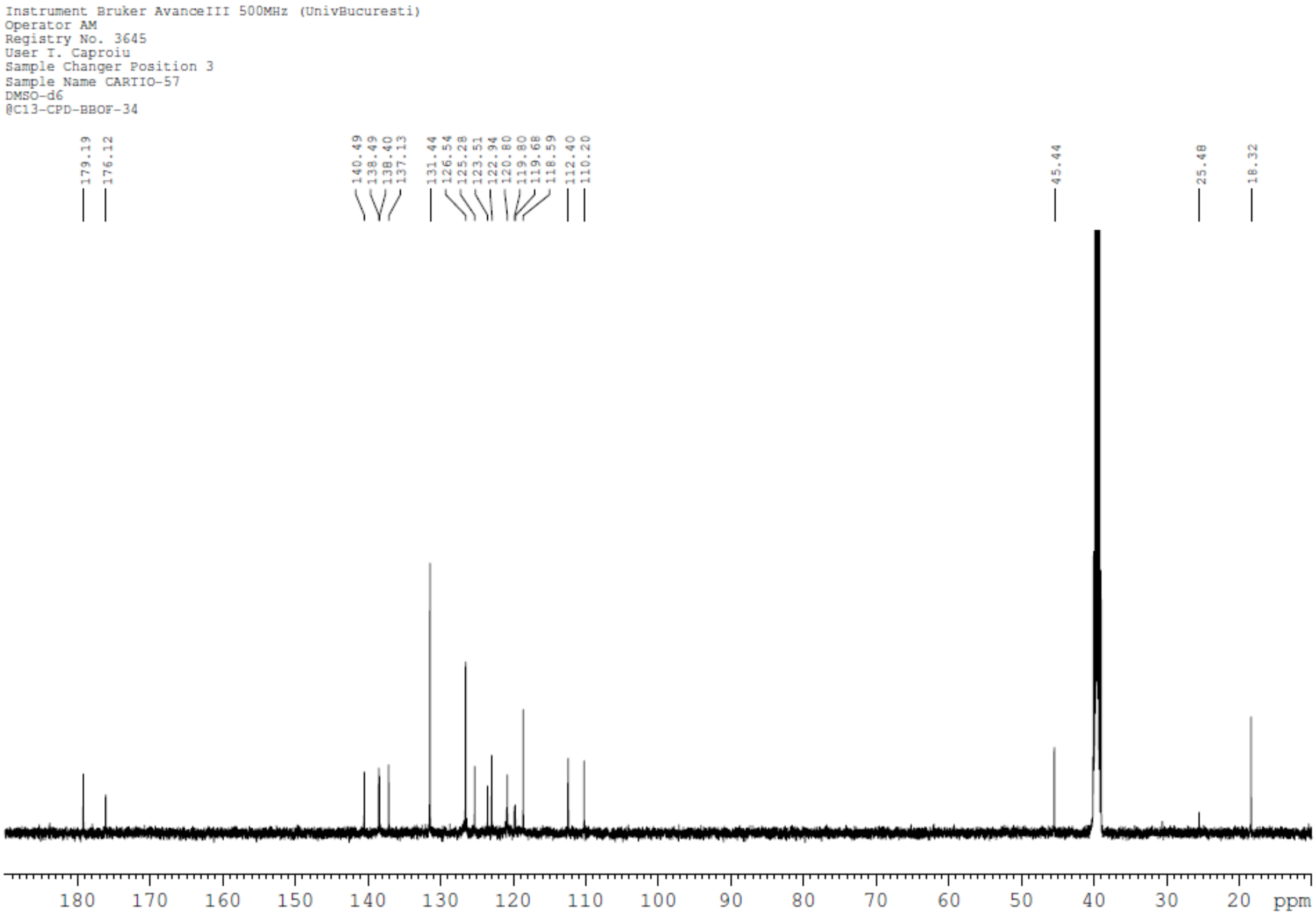

Figure S13. The ^{13}C-NMR spectrum of compound **1c**

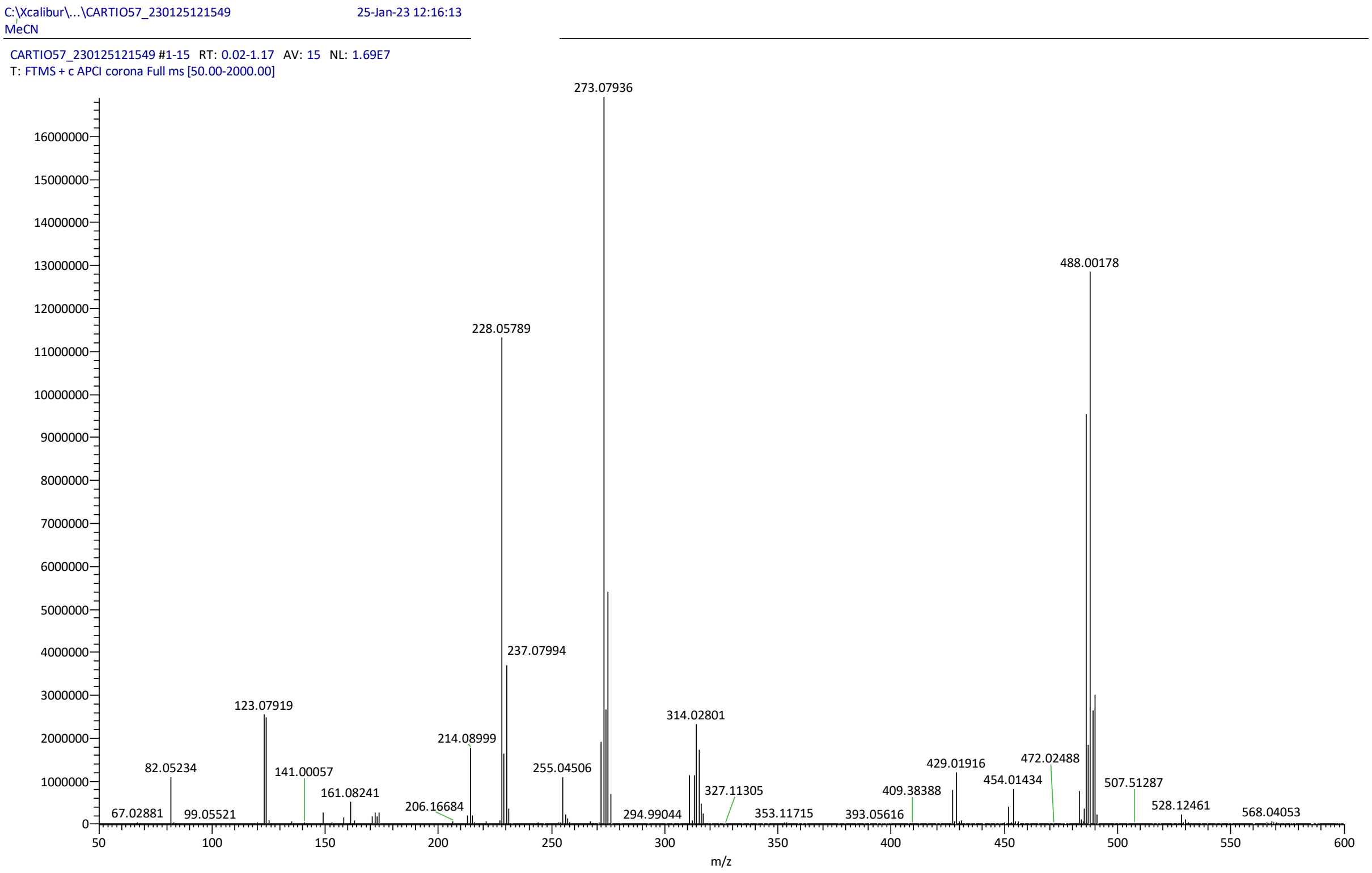

Figure S14. The APCI+ MS spectrum of **1c** in MeCN

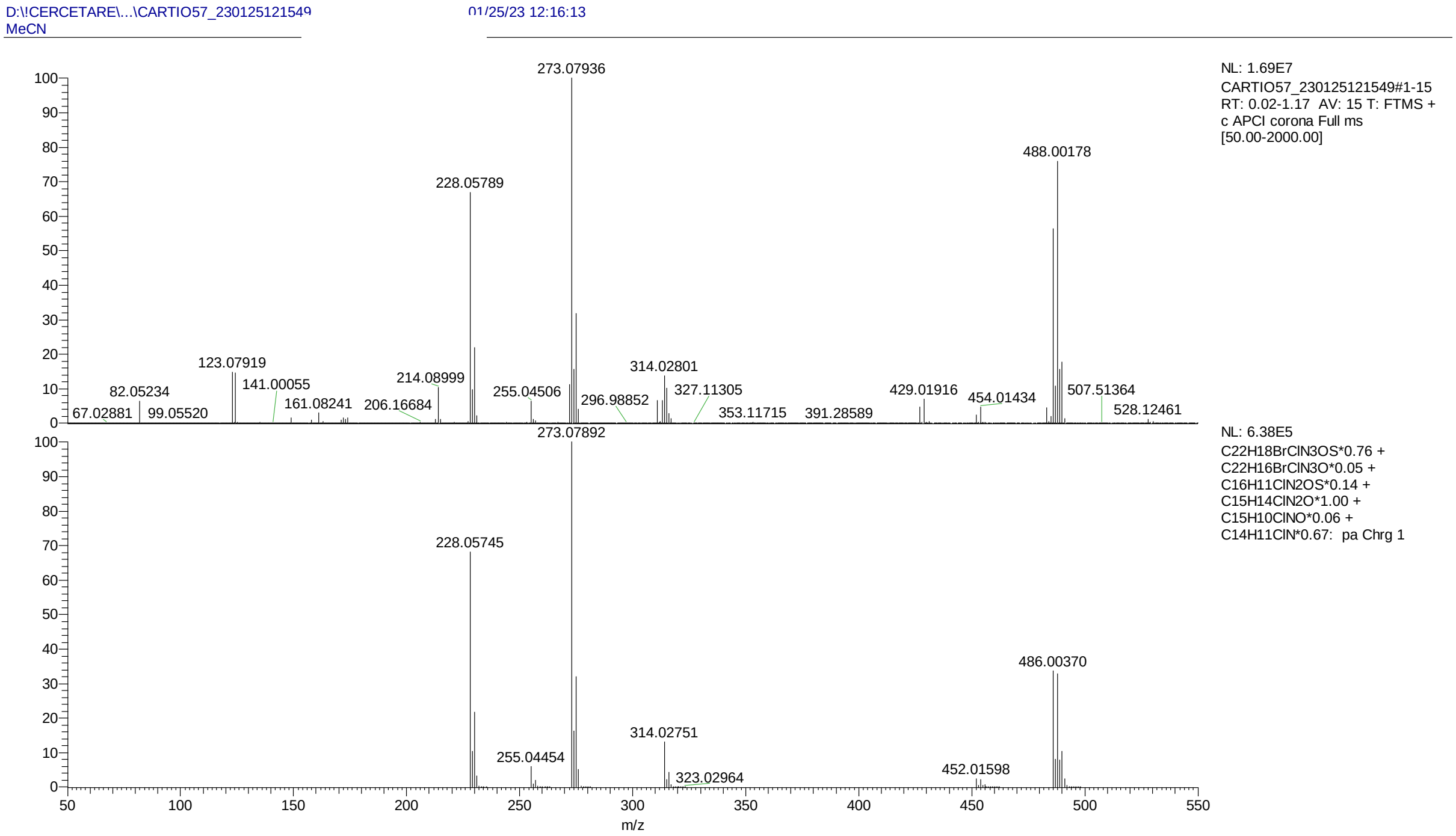

Figure S15. The experimental (up) and calculated (down) APCI+ MS spectra of **1c**

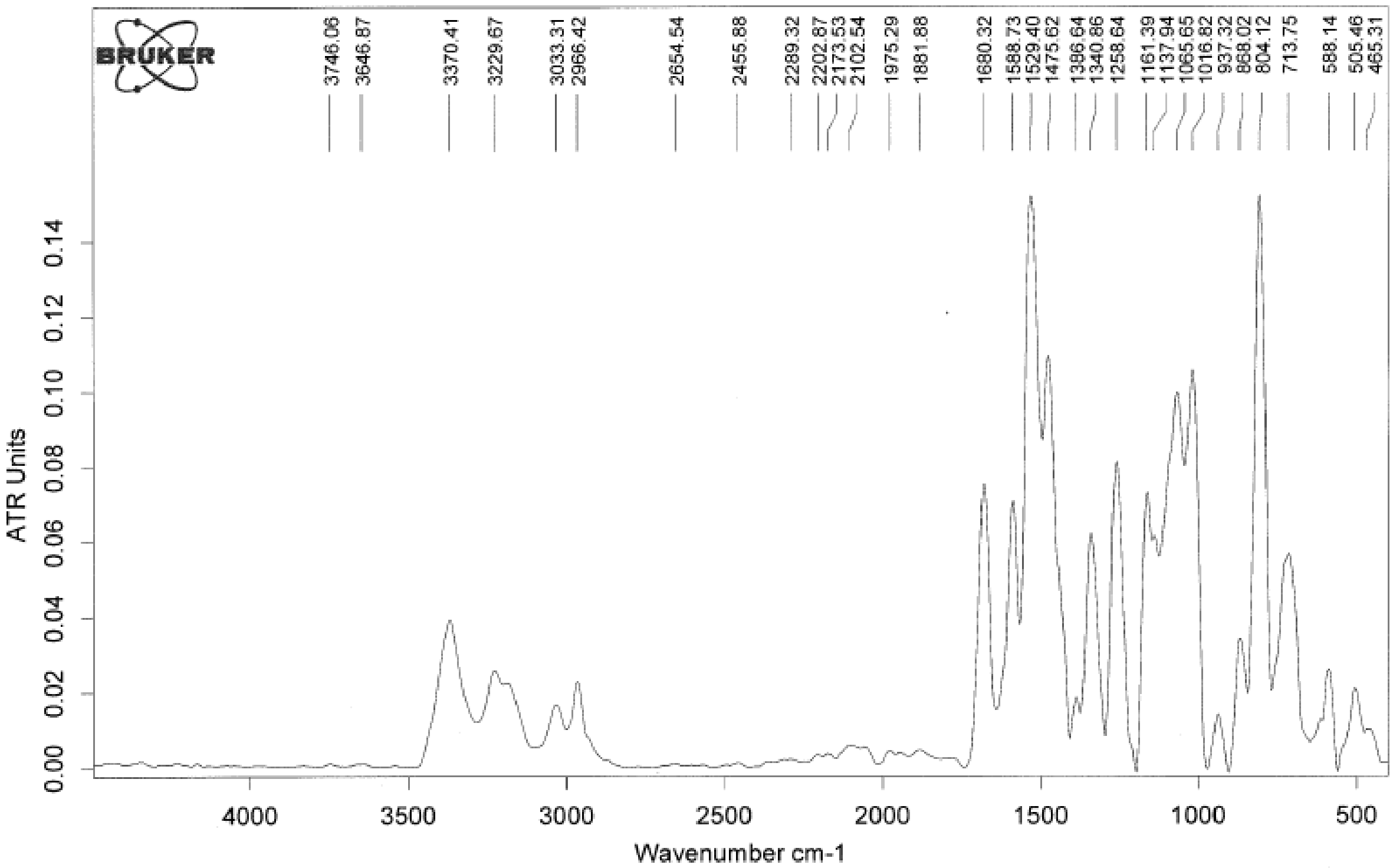

Figure S16. The IR spectrum of compound **1d**

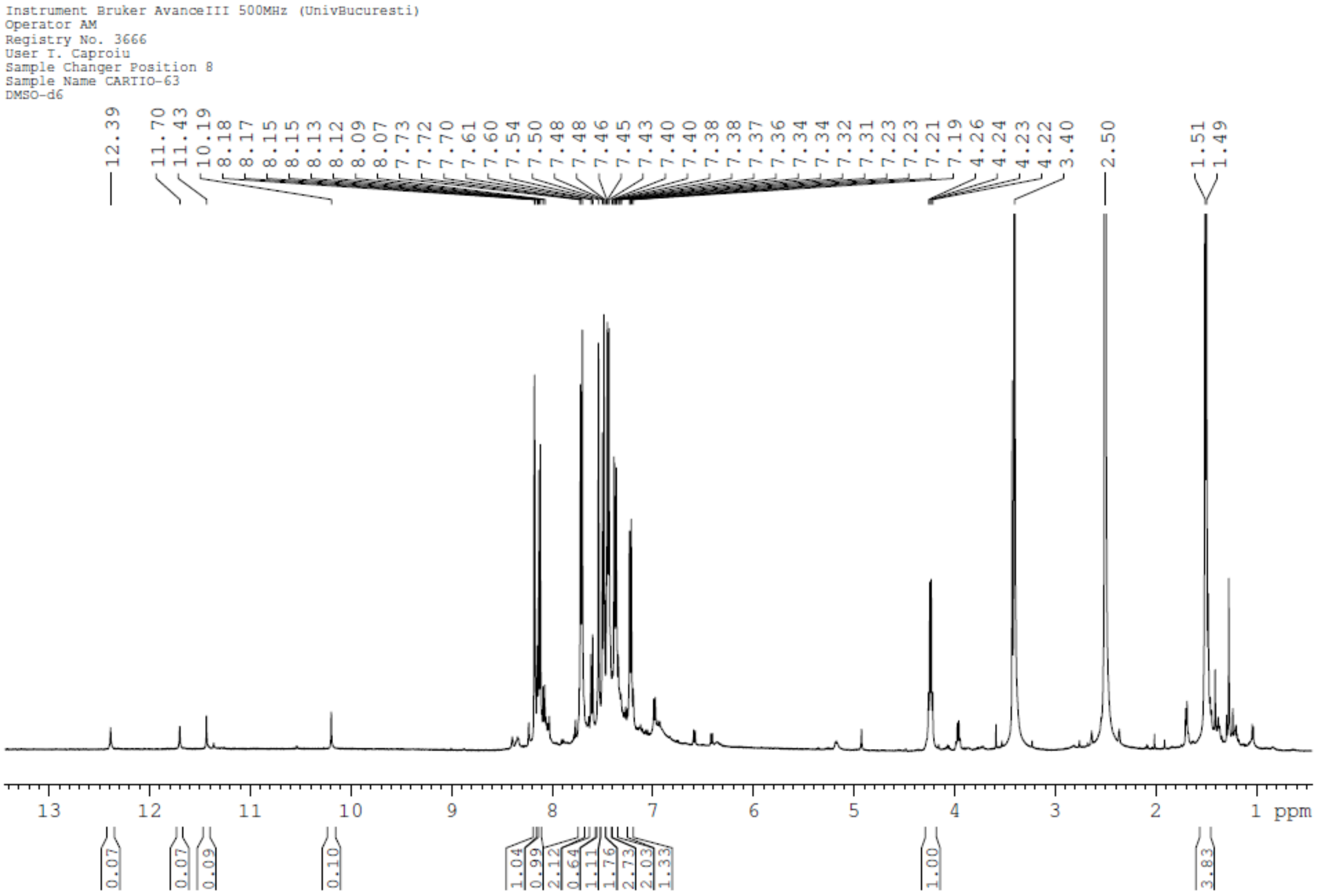

Figure S17. The ^{1}H-NMR spectrum of compound **1d**

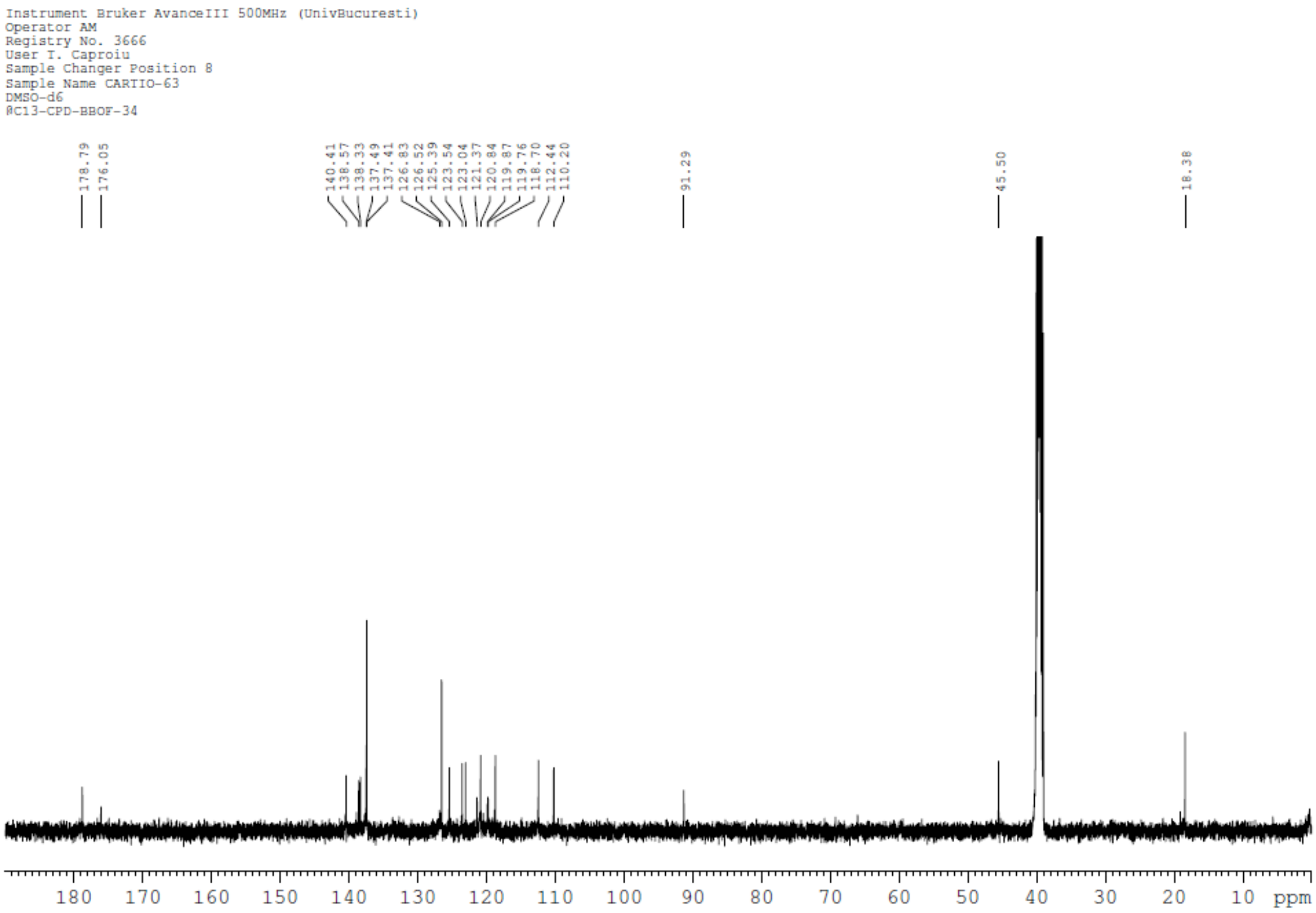

Figure S18. The ^{13}C-NMR spectrum of compound **1d**

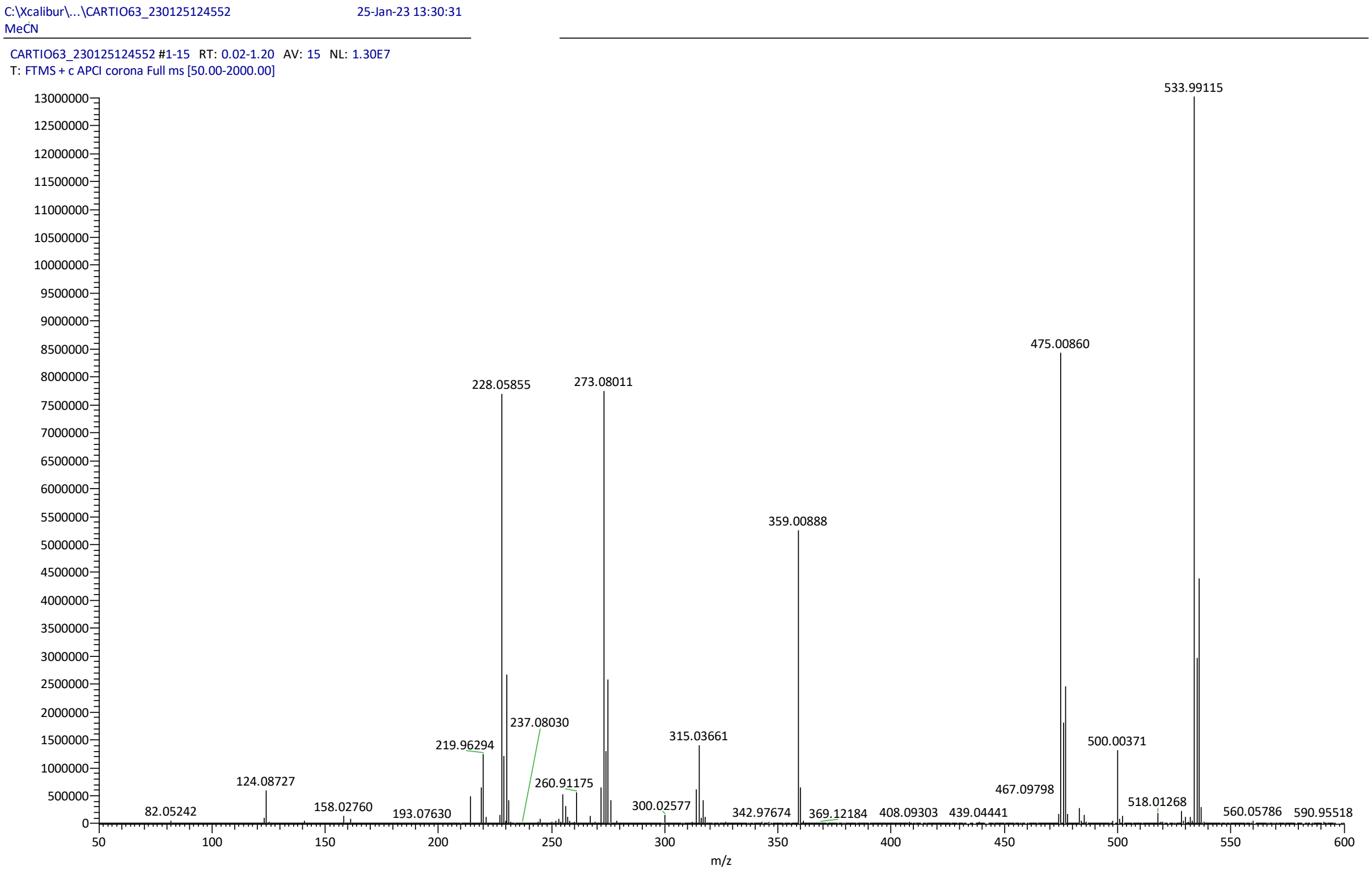

Figure S19. The APCI+ MS spectrum of **1d** in MeCN

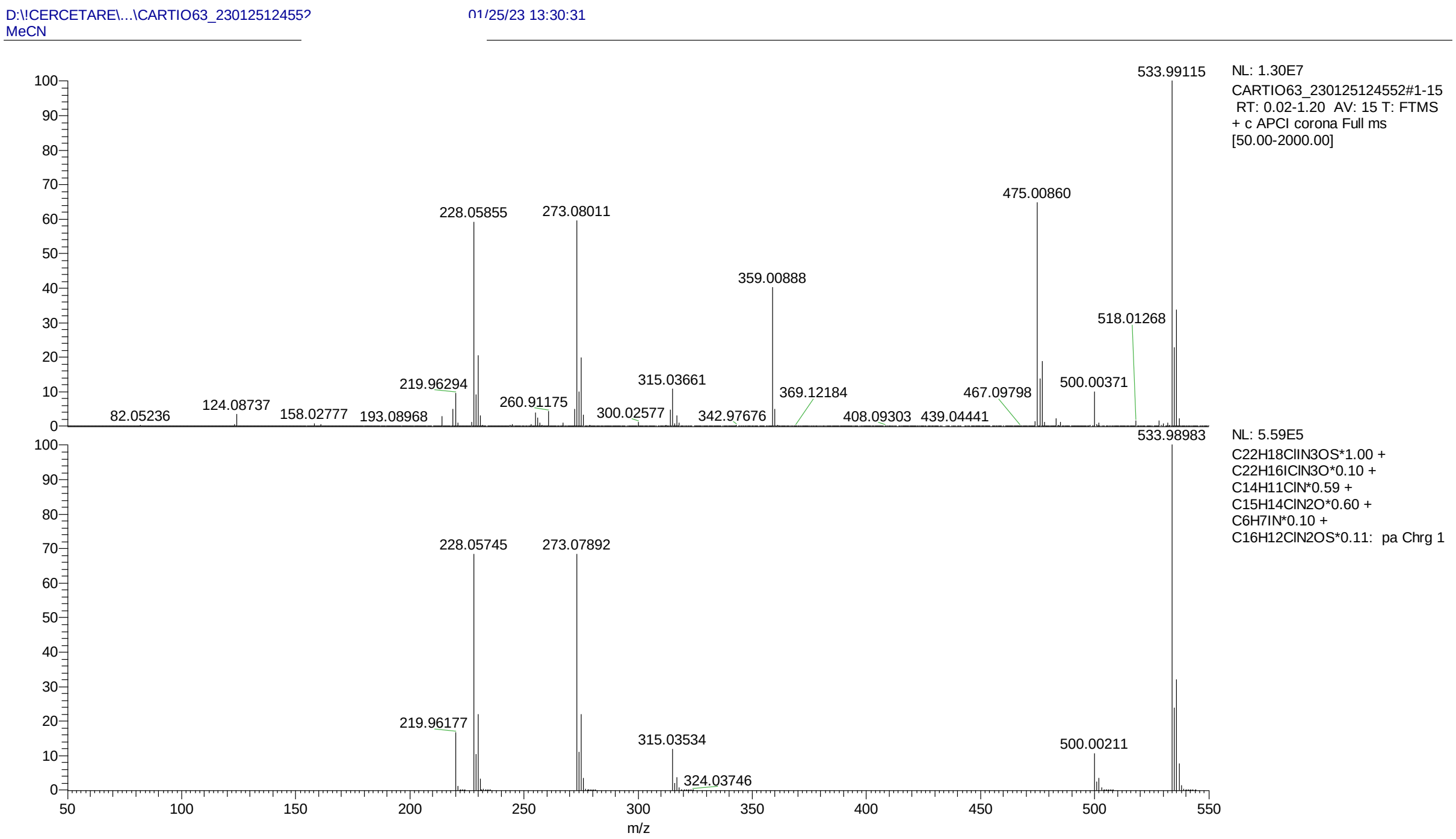

Figure S20. The experimental (up) and calculated (down) APCI+ MS spectra of **1d**

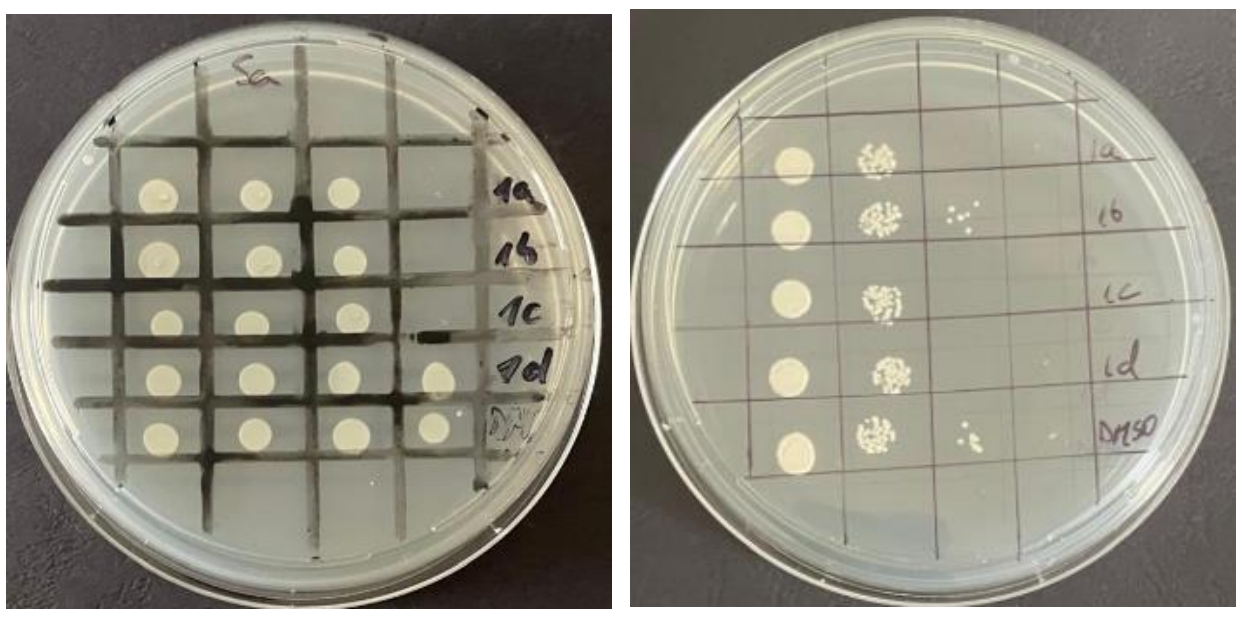

Figure S21. Representative agar plates showing microbial survival following exposure to different concentrations of the tested compounds, used for minimum microbicidal concentration (MMC) determination (left – *S. aureus* and right – *C. albicans*).

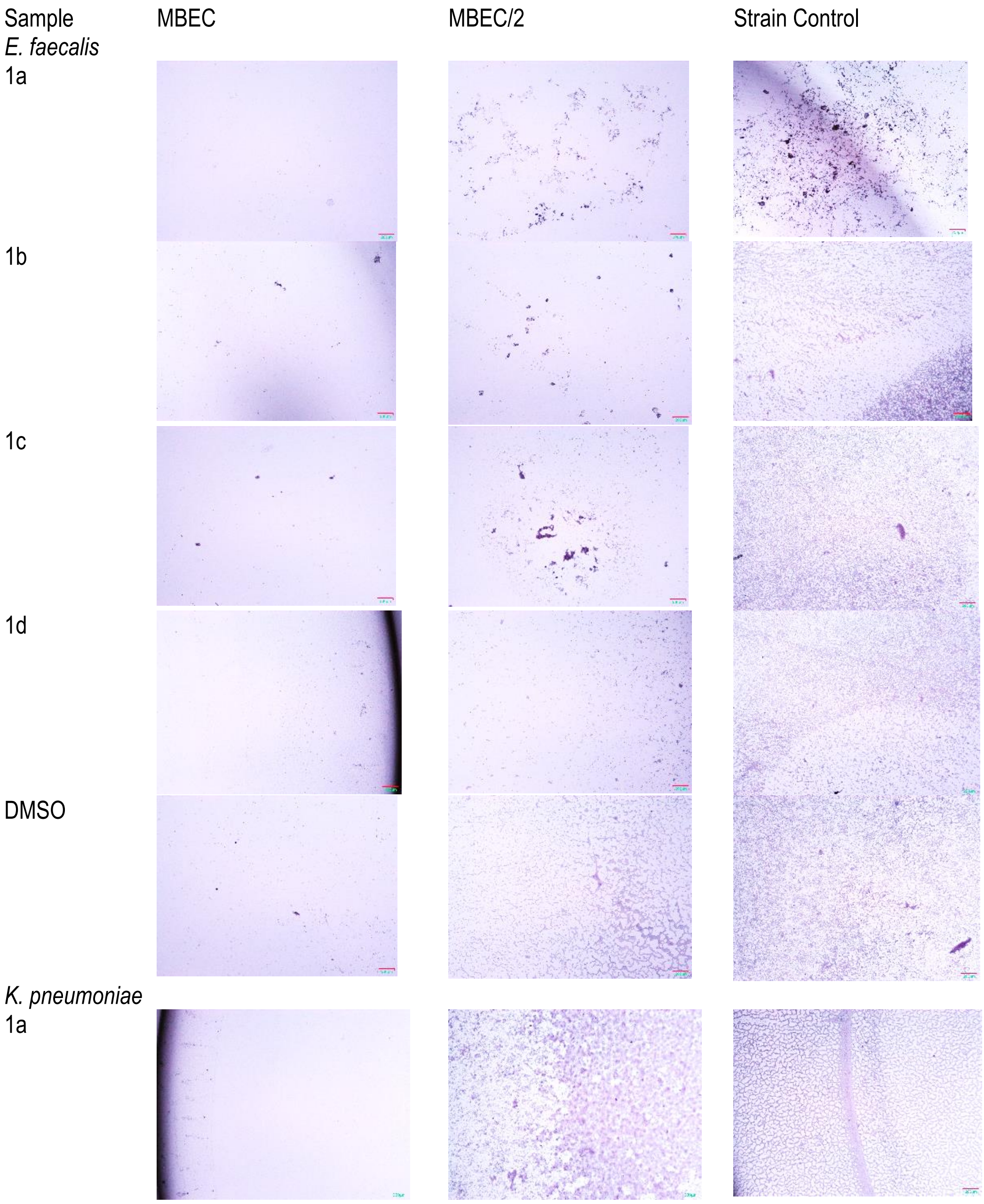

1b

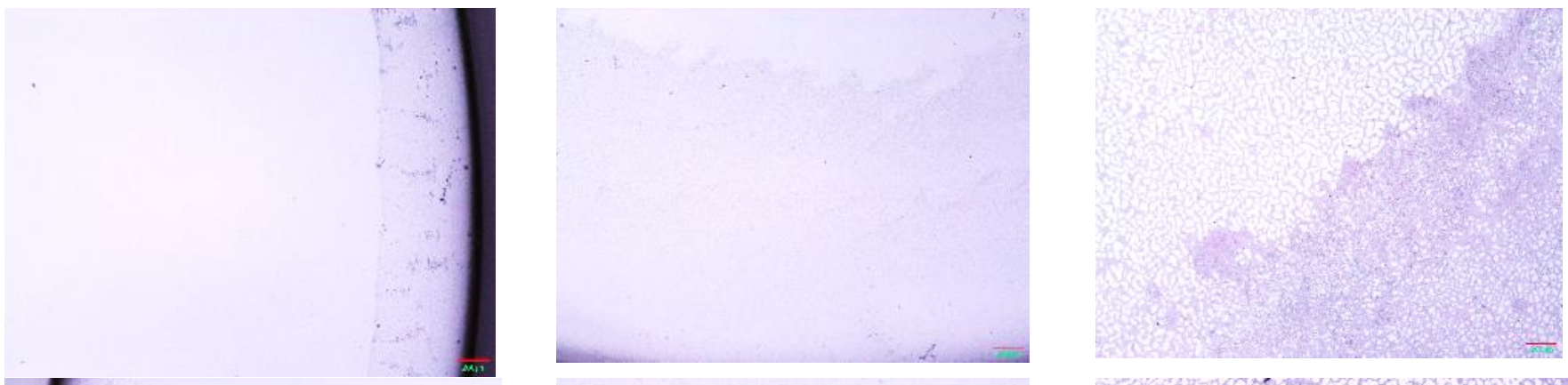

1c

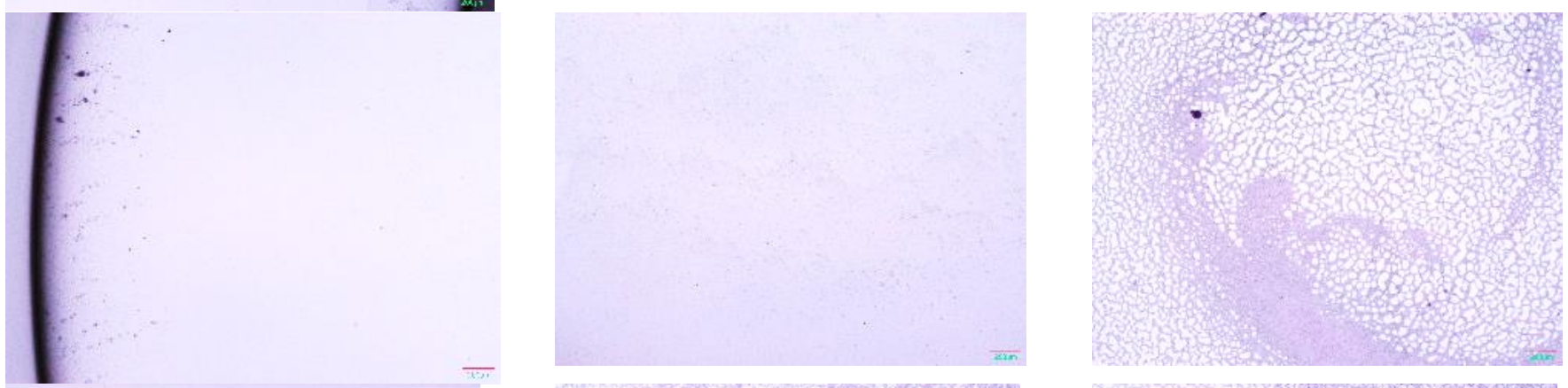

1d

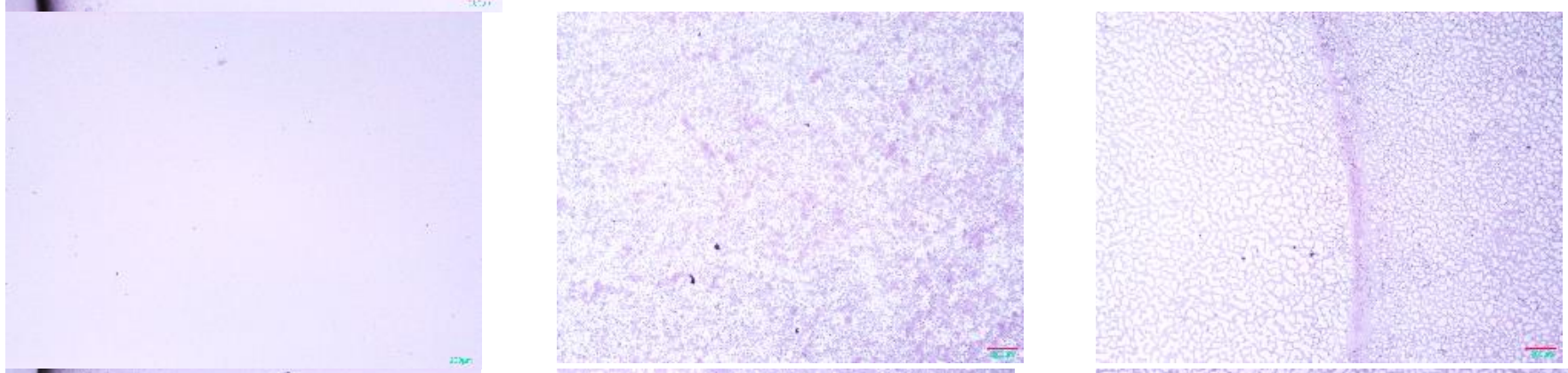

DMSO

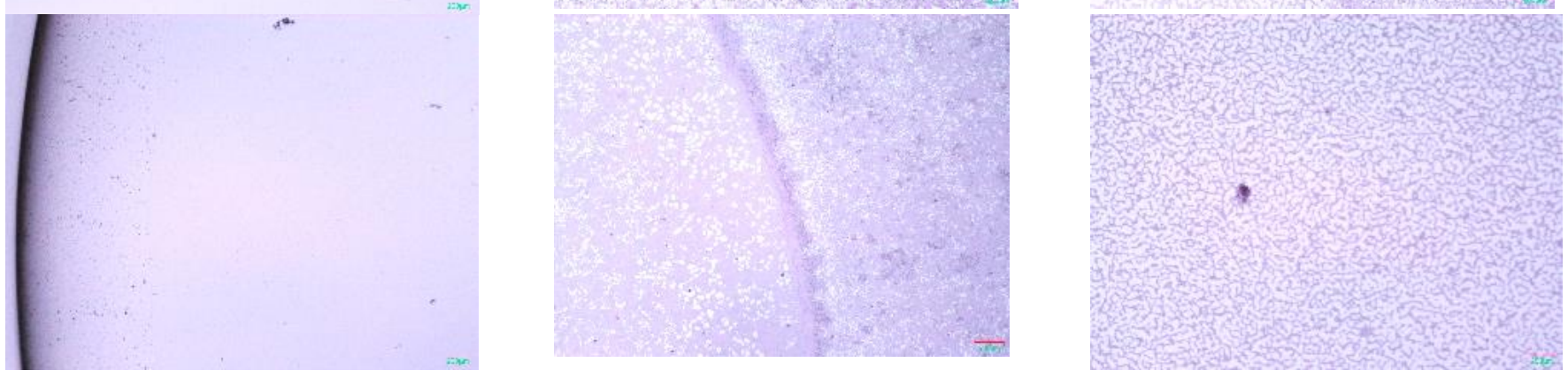

C. albicans

1a

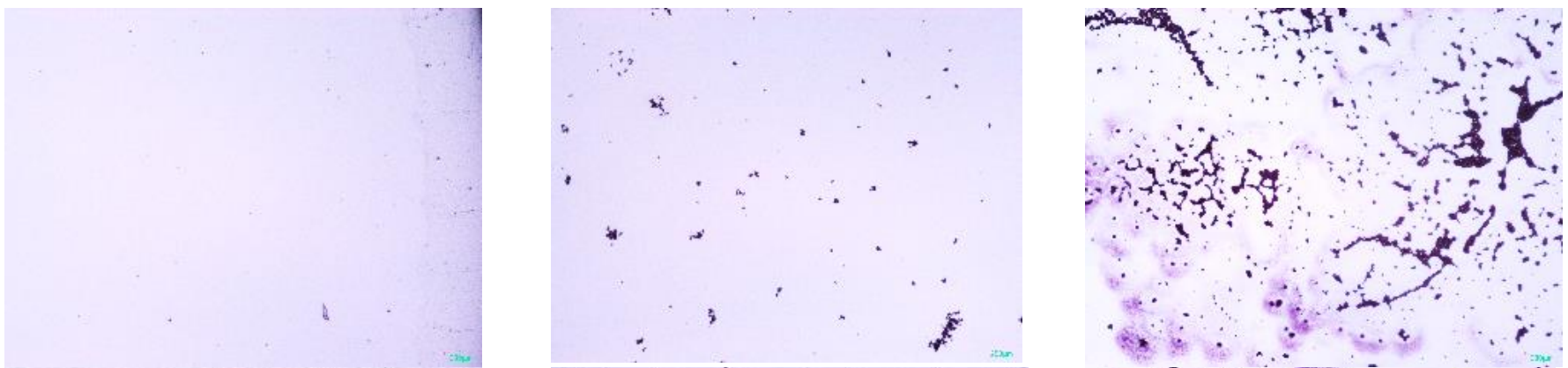

1b

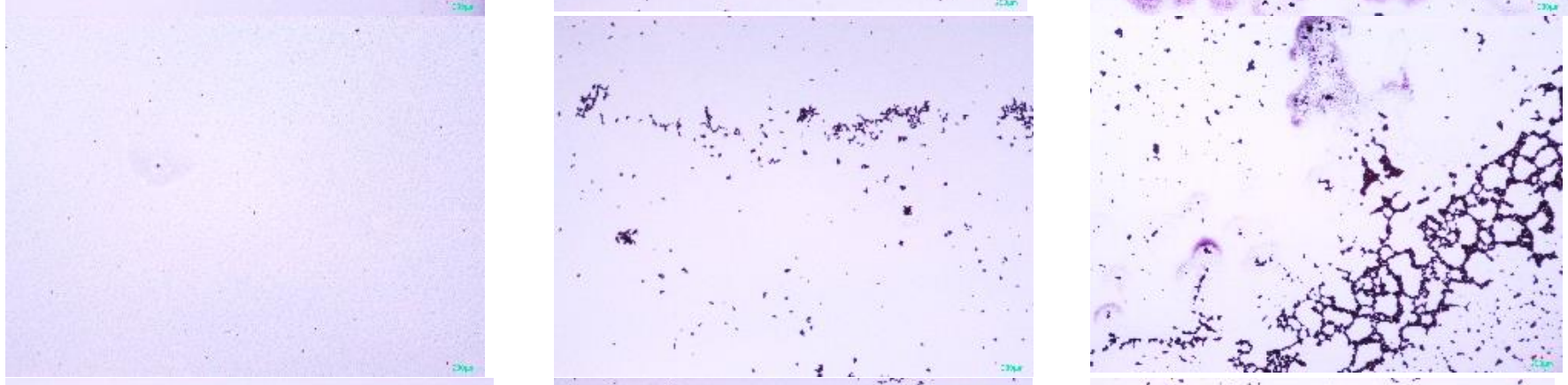

1c

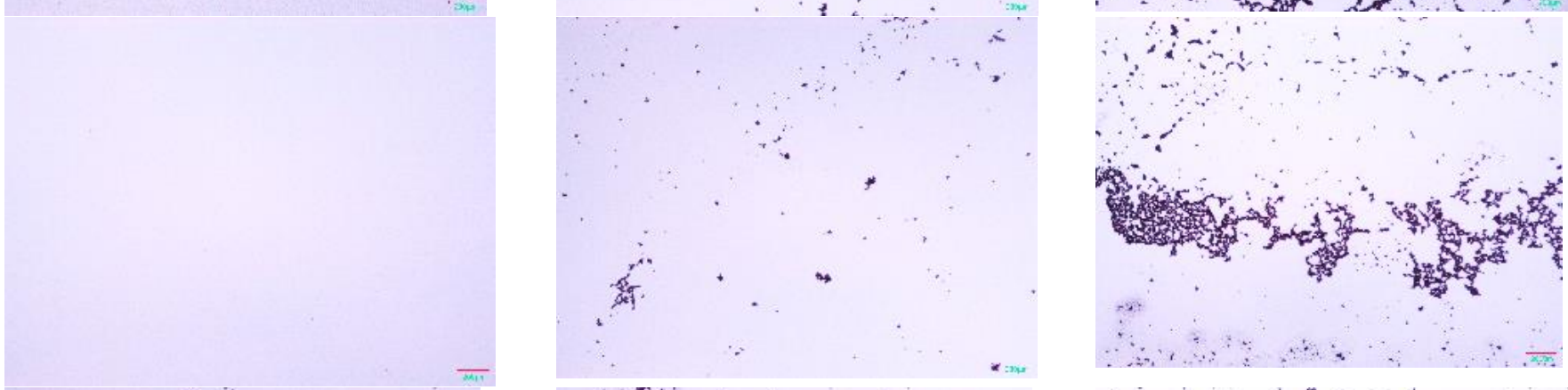

1d

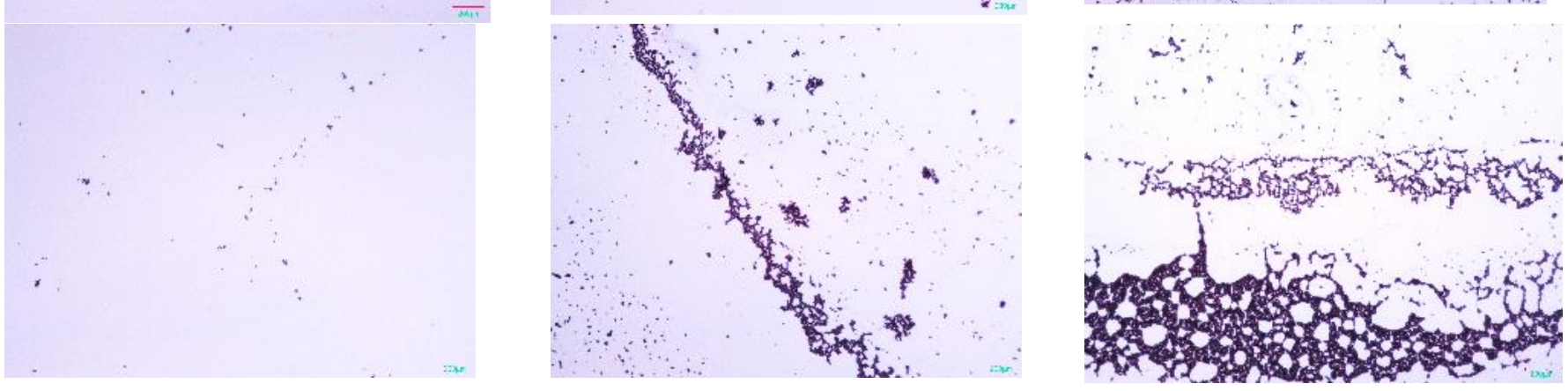

DMSO

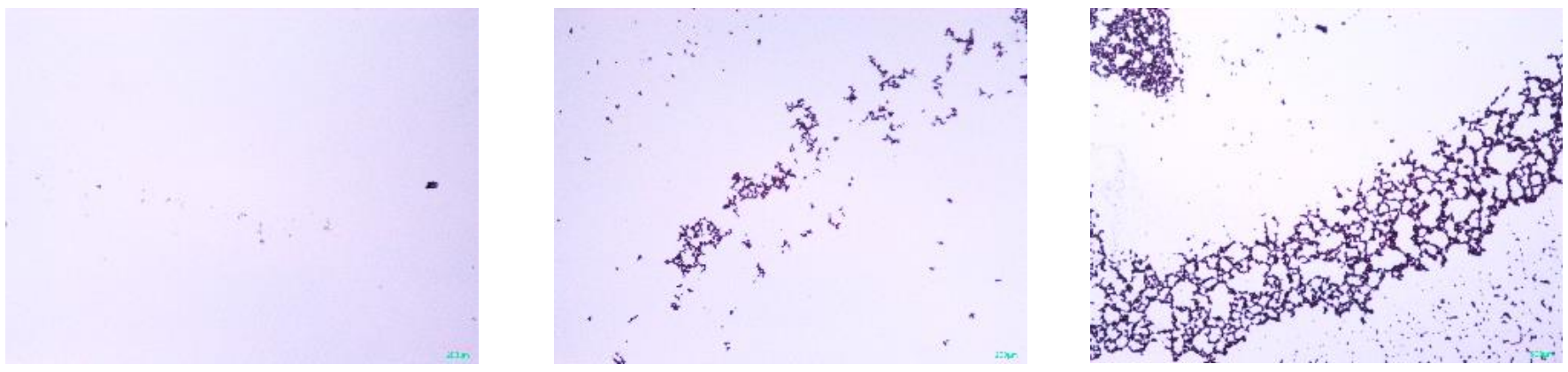

Figure S22**.** Microscopic evaluation of the adherence to an inert substrate of *E. faecalis*, *K. pneumoniae*, and *C. albicans* cells after exposure to MBEC and MBEC/2 concentration, compared to the untreated control (strain control).
